# Dramatic changes in gene expression in different forms of *Crithidia fasciculata* reveal potential mechanisms for insect-specific adhesion in kinetoplastid parasites

**DOI:** 10.1371/journal.pntd.0007570

**Published:** 2019-07-29

**Authors:** John N. Filosa, Corbett T. Berry, Gordon Ruthel, Stephen M. Beverley, Wesley C. Warren, Chad Tomlinson, Peter J. Myler, Elizabeth A. Dudkin, Megan L. Povelones, Michael Povelones

**Affiliations:** 1 Department of Pathobiology, University of Pennsylvania School of Veterinary Medicine, Philadelphia, Pennsylvania, United States of America; 2 Department of Molecular Microbiology, Washington University School of Medicine, St. Louis, Missouri, United States of America; 3 University of Missouri, Bond Life Sciences Center, Columbia, Missouri, United States of America; 4 McDonnell Genome Institute, Washington University School of Medicine, St. Louis, Missouri, United States of America; 5 Center for Global Infectious Disease Research, Seattle Children’s Research Institute, Seattle, Washington, United States of America; 6 Department of Global Health, University of Washington, Seattle, Washington, United States of America; 7 Department of Biomedical Informatics and Medical Education, University of Washington, Seattle, Washington, United States of America; 8 Department of Biology, Penn State Brandywine, Media, Pennsylvania, United States of America; Liverpool School of Tropical Medicine, UNITED KINGDOM

## Abstract

Kinetoplastids are a group of parasites that includes several medically-important species. These human-infective species are transmitted by insect vectors in which the parasites undergo specific developmental transformations. For each species, this includes a stage in which parasites adhere to insect tissue via a hemidesmosome-like structure. Although this structure has been described morphologically, it has never been molecularly characterized. We are using *Crithidia fasciculata*, an insect parasite that produces large numbers of adherent parasites inside its mosquito host, as a model kinetoplastid to investigate both the mechanism of adherence and the signals required for differentiation to an adherent form. An advantage of *C*. *fasciculata* is that adherent parasites can be generated both *in vitro*, allowing a direct comparison to cultured swimming forms, as well as *in vivo* within the mosquito. Using RNAseq, we identify genes associated with adherence in *C*. *fasciculata*. As almost all of these genes have orthologs in other kinetoplastid species, our findings may reveal shared mechanisms of adherence, allowing investigation of a crucial step in parasite development and disease transmission. In addition, dual-RNAseq allowed us to explore the interaction between the parasites and the mosquito. Although the infection is well-tolerated, anti-microbial peptides and other components of the mosquito innate immune system are upregulated. Our findings indicate that *C*. *fasciculata* is a powerful model system for probing kinetoplastid-insect interactions.

## Author summary

Kinetoplastids are single-celled parasites that cause devastating human diseases worldwide. Although this group includes many species that infect a variety of hosts, they have a great deal of shared biology. One relatively unexplored aspect of the kinetoplastid life cycle is their ability to adhere to insect tissue. For pathogenic species, adherence is critical for transmission by insect vectors. We have used an insect parasite called *Crithidia fasciculata* as a model kinetoplastid to reveal shared mechanisms of insect adherence. We have compared gene expression profiles of motile, non-adherent *C*. *fasciculata* to those of *C*. *fasciculata* adhered to non-living substrates and those attached to the hindgut of mosquitoes. Through this analysis, we have identified a large number of candidate proteins that may mediate adhesion in these and related parasites. In addition, our findings suggest that the mosquito immune system is responding to the presence of parasites in the gut. These results establish a new, robust system to explore the interaction between kinetoplastids and their insect hosts.

## Introduction

Kinetoplastids are eukaryotic parasites, some of which are agents of important tropical diseases such as Human African Trypanosomiasis, Chagas Disease, and Leishmaniasis. Insects serve as hosts and vectors for human-infective kinetoplastids. While the cell biology of these parasites has been studied intensively for decades, many of the life cycle stages that develop in the insect vector are the least well characterized. This is because certain developmental forms are not amenable to cultivation and their abundance or location in the insect complicates their isolation and analysis. In addition, some vector species can be difficult to maintain, and it can be challenging to achieve robust and reliable laboratory infections.

Kinetoplastids colonize different insect vectors and have distinct life cycles within those vectors. *Leishmania* spp. are transmitted by phlebotomine sandflies. With the exception of members of the *Vianna* subgenus, such as *L*. *braziliensis*, all *Leishmania* species undergo a similar developmental program within the fly [1]. They enter during blood feeding and proliferate in the midgut as promastigotes, which divide as rosettes with their flagella oriented towards the center [2, 3]. As the bloodmeal is digested, promastigotes transform into motile nectomonads, which attach to epithelial cells via their flagellum. Epithelial attachment is required to maintain the parasites in the gut during blood meal processing [4, 5]. Nectomonads then migrate anteriorly and attach to the stomodeal valve where they differentiate into haptomonads and eventually metacyclic promastigotes. Accumulating parasites compromise the function of the valve, allowing parasites to migrate beyond it and enter the proboscis where they can be transmitted during the next bloodmeal [3].

Upon ingestion by the tsetse fly during blood feeding, *Trypanosoma brucei* differentiates in the fly midgut into replicating promastigotes. These migrate from the midgut to the salivary glands where they adhere to the salivary epithelium [6]. Here, they form transmission-stage metacyclics that can be introduced into a mammalian host during blood feeding [7]. *Trypanosoma cruzi* is transmitted by reduviid bugs. Epimastigotes first adhere to the posterior portion of the midgut and then to the hindgut and rectal epithelia. They then transform into metacyclics, which are defecated onto the host during insect blood feeding [8]. Adherence to the gut is not only important for *T*. *cruzi* maintenance, it is also a crucial step in the differentiation of epimastigotes to infective metacyclics [9].

One consistent feature in the life cycle of all these pathogenic kinetoplastids is that they adhere via their flagellum to insect tissue. Adherence is crucial for vector colonization, parasite differentiation, and parasite transmission [6, 9, 10]. Furthermore, the ultrastructural nature of this attachment is remarkably similar among the different species, resembling an electron-dense, hemidesmosome-like plaque connecting the flagellum of the parasite with the insect tissue [11, 12]. While a handful of molecules have been proposed to play a role in adhesion [13–18], the molecular composition of the attachment structure has yet to be described in detail.

In addition to pathogenic species, there are monoxenous kinetoplastids that exclusively parasitize insects, such as *Crithidia fasciculata*, which is a parasite of mosquitoes [19]. Large quantities of *C*. *fasciculata* can be grown rapidly and inexpensively, making them a useful model kinetoplastid for biochemical and molecular studies. *C*. *fasciculata* can effectively colonize a range of mosquito species, adhering to the hindgut and rectal papillae of the mosquito [20]. Like other kinetoplastids, adhesion is mediated by the flagellum, and the adhesive structure resembles a hemidesmosome [21, 22]. Adherent *C*. *fasciculata*, called haptomonads, are rounder than the swimming nectomonad form and have a dramatically shortened flagellum. Interestingly, *C*. *fasciculata* cells can form similar attachments to non-living substrates, including tissue culture plastic [23, 24].

Given the importance of parasite adherence to insect tissue in the transmission of pathogenic kinetoplastids, and the likelihood that all kinetoplastids may have evolved similar mechanisms for this process, we have used *C*. *fasciculata* to investigate the cell and molecular biology of kinetoplastid adhesion. We compared the transcriptomic profiles of swimming and adherent *C*. *fasciculata* cells grown in culture. We also characterized the transcriptomic profile of *C*. *fasciculata* isolated from the hindgut of infected *Aedes aegypti* mosquitoes. Although parasites recovered from mosquitoes were transcriptionally distinct from both cultured forms, they most closely resemble adherent cells and share a subset of upregulated genes with putative roles in adhesion. We additionally analyzed mosquito samples for changes in gene expression following infection with *C*. *fasciculata* and identified a small number of differentially regulated genes indicating that the mosquito is responding to the parasites. Taken together, our data provide a detailed analysis of *C*. *fasciculata* growth *in vitro* and *in vivo*, and suggest that adherent *C*. *fasciculata* may be better-suited for survival and proliferation in the mosquito host, while swimmers may be better adapted for dispersal and survival while awaiting uptake by a mosquito.

## Methods

### Cell culture

The CfC1 genome reference strain of *Crithidia fasciculata* was used for our experiments. The line was obtained from the laboratory of Dr. Stephen Beverley (Washington University). For these experiments, cells were maintained in complete brain heart infusion medium (BHI) made by dissolving 37 g of powder (Sigma) per liter of water and supplementing with bovine hemin (Sigma) to a final concentration of 20 μg/ml. Cells were passaged every 2–3 days and cultured between 27–28 °C at a density of 10^5^−10^8^ cells/ml in non-treated tissue culture flasks. For cultures of swimming cells, flasks were placed on a rocker, while flasks for adherent cells were left stationary on the incubator shelf. All washes of adherent cultures were performed by pipetting the wash solution directly onto the flask surface, then rocking the flask. Swimming cells were counted by fixing in 0.3% formalin followed by staining with Gentian violet (Harleco) in 0.2 M NaCl, 0.7 mM EDTA. Fixed, stained cells were then counted on a hemocytometer.

### Mosquito infection

*Aedes aegypti* Liverpool strain (LVP-IB12) were used for these experiments. This colony is maintained under standard laboratory conditions from eggs obtained from BEI Resources, NIAID, NIH (MRA-735) contributed by David W. Severson. To infect mosquitoes with *C*. *fasciculata*, 10^8^ swimming cells from a log-phase culture were pelleted by centrifugation at 1000 rcf. After removing the medium, the cell pellet was resuspended in a solution containing 4 ml PBS and 8 ml 10% sucrose. This solution was used to saturate four cotton balls, which were divided between two containers each with approximately 50 female *Ae*. *aegypti* 3–6 days post-eclosion. Prior to infection, mosquitoes were starved overnight. Mosquitoes were then allowed to feed on *C*. *fasciculata*/sucrose-saturated cotton balls for 24 h before being switched back to 10% sucrose. Seven days after feeding, mosquito hindguts and rectal papillae were dissected in PBS (removing the midgut and Malpighian tubules). At least five mosquitoes from each cup were examined for infection prevalence, which is typically 100%. The remainder were transferred to Trizol and frozen for RNA isolation. For survival analysis, male and female *Ae*. *aegypti* 6–8 days post-eclosion were placed separately into 4 cups and starved for 24 h. Control and *C*. *fasciculata* infection treatments were performed as described above. 80 mosquitoes were used for each of the 4 groups. Mosquito mortality was recorded daily. Final counts were made 28 days after treatment. Kaplan-Meier plots were generated and analyzed using Prism (GraphPad).

### Plasmids

To create GFP-expressing *C*. *fasciculata*, we used the pNUS series of plasmids that were created for use in *C*. *fasciculata* and are maintained in transformed cells as episomes [25]. To increase expression of the fluorescent protein, an rDNA promoter was introduced [26, 27]. The rDNA promoter was PCR amplified and inserted into the HindIII site upstream of the GFP expression cassette. Plasmids were electroporated into *C*. *fasciculata* CfC1 cells with a BTX-ECM600 device as described [28].

### Imaging cultured rosettes

To image rosettes in culture, cells were adhered to poly-L-lysine-coated glass coverslips in 6-well culture dishes for two hours in BHI. Coverslips were gently washed three times with PBS. BHI was then added back to the wells, and plates were incubated for approximately 24 h to allow for growth of adherent cells. The coverslip was removed, mounted on a glass slide, and imaged. To image swimming cells, a sample was removed from culture, placed on a charged glass slide, covered with a coverslip, and imaged. Live-cell imaging was performed using a Zeiss Axioscope.A1 upright LED fluorescence microscope equipped with a Zeiss AxioCam ICm1 camera. For time-lapse imaging of rosettes, cells were adhered to poly-L-lysine-treated Mattek dishes for 2 h, followed by 3 PBS washes to remove any swimming cells. After the PBS washes, a small volume of medium was added back to the dish into the well formed by the Mattek coverslip. An additional glass coverslip was sealed over the cells to prevent evaporation and provide the optical clarity required for optimal imaging. The adhered cells were imaged on a Leica DMI4000 spinning disk confocal microscope. Multiple positions were imaged every 2 min.

### RNA isolation

To isolate RNA from swimming cells, cultures were established with 10^8^ cells in 10 ml of BHI in a 25 cm^2^ flask and allowed to grow on a rocker at 27 °C for 24 h. To generate samples enriched for adherent cells, 2 x 10^8^ cells were diluted in 20 ml of BHI in a 75 cm^2^ flask. These flasks were incubated for 2 h on the incubator shelf to allow cells to adhere. The culture medium was then removed and flasks were washed twice with 20 ml BHI medium to remove non-adherent cells. BHI medium (20 ml) was added back to the flasks, which were then incubated for 24 h on the incubator shelf. Adherent cells were lysed in the flask by removing culture medium, washing three times with BHI to remove swimming cells, then adding 4 ml Trizol (ThermoFisher) directly to the flask. For swimming cultures, 7 x 10^8^ cells were centrifuged at 1000 rcf for 10 min. The supernatant was removed and the cell pellet resuspended in 2 ml Trizol. Total RNA was isolated according to the manufacturer’s protocol for cells grown in suspension. RNA pellets were resuspended in 40 μl water and stored at -80 °C. For mosquito hindgut samples, 30 hindguts from each condition (uninfected and infected) were stored at -80 °C in 500 μl Trizol and 100 μl glass beads and processed together for RNA isolation. Hindgut samples were supplemented with 120 μl of chloroform and then homogenized using a FastPrep-24 at 4.0 m/s for 20 s. Following homogenization, total RNA was isolated following the standard protocol. RNA pellets were resuspended in 40 μl water and stored at -80 °C. Contaminating DNA was removed from RNA samples by treating them with Turbo DNA-free (ThermoFisher).

### qPCR analysis

Total RNA samples were purified from cultured adherent and swimming *C*. *fasciculata* as well as from infected and uninfected hindguts as described above with the following modifications. *Ae*. *aegypti* hindgut samples were processed using Direct-zol RNA MicroPrep columns (Zymo Research) according to the manufacturer’s directions including an on-column DNase treatment step. After purification, 1 μg of total RNA was reverse transcribed into cDNA using iScript (Bio-Rad) according to the manufacturer’s protocol. Quantitative PCR was performed with PerfeCTa SYBR Green Supermix (QuantaBio) on a ViiA 7 system (Applied Biosystems).

### Preparation of libraries for RNAseq

RNA quality was evaluated using an Agilent 4200 TapeStation. RNA concentration was determined by Qubit 3 Fluorometer (ThermoFisher). Libraries from triplicate cultured swimming, cultured adherent, uninfected, and infected *Ae*. *aegypti* hindguts were prepared from 1 μg of RNA using an Illumina TruSeq stranded mRNA library kit according to the manufacturer’s instructions. Libraries were pooled and sequenced in a single run on an Illumina NextSeq 500 sequencer. Single-end reads (75 bp) were generated and 600 million reads passed the quality filter.

### Genome sequencing and assembly

The *Crithidia fasciculata* (strain/isolate Cf-C1) was obtained from Dr. Larry Simpson, University of California, Los Angeles. Genomic DNA was prepared by CsCl/Ethidium bromide density gradient centrifugation to minimize the amount of kinetoplastid (mitochondrial) DNA. A total of 12 single molecule real-time (SMRT) cells of sequence data were generated on the PacBio RS II instrument, comprised of SMRT cells of 10Kb library fragment size utilizing the P4/C2 chemistry with a post-filtered yield of 1.6 Gb of data (48X) and eight SMRT cells of 20Kb library data utilizing the P5/C3 chemistry for a filtered yield of 2.6 Gb of data (77X). All SMRT reads were assembled using HGAP [29] and ECtools [30]. HGAP entails a three-part process starting with error correction of the reads during the initial preassembly stage. Preassembly is accomplished by aligning SMRT reads to seed reads and outputting a consensus of the aligned reads resulting in long, accurate fragments of the genome. The second step of HGAP involves performing an assembly of the preassembled reads using the Celera assembler [31]. The ECtools error correction used the assembled unitig or contig FASTA formatted sequences for error correction of the SMRT sequences. This resulted in 49.5X coverage of error-corrected sequences to use for the primary assembly. Several minimum seed read lengths were tested to derive the optimal combination of contig length and genome assembly size. Consensus base error correction on the assembled contigs were accomplished with Quiver, a quality-aware consensus algorithm which uses quality values encoded in the primary bax.h5 file format to output a highly accurate consensus. For the purpose of building pseudochromosomes we used Nucmer/Promer and a series of custom scripts to align the draft assembly contigs to the chromosomal build of *Leishmania major* Friedlin. To improve upon the initial chromosomal build, we used the ABACAS software [32]. ABACAS uses MUMmer to determine alignment position and synteny of assembled contigs versus a reference, resulting in the output of a new consensus FASTA sequence of ordered, oriented contigs along the queried reference. Separate ABACAS jobs were performed on each of the 31 chromosome sequences from the chromosomal build in order to integrate the assembled contigs. PBJelly (v14.9.9) [33] was then used to extend and fill gaps within the pseudoautosomal gap regions. Since PBJelly integrates error-prone SMRT sequences into the assembly gaps, we ran two iterations of iCORN [34] that utilizes highly accurate aligned Illumina data to correct small assembly base insertions and deletions genome-wide. The final assembly, denoted *Crithidia fasiculata*-14.0, was submitted to GenBank and assigned accession number GCA_000331325.2. Annotations were generated using the Sanger annotation platform [35] and through manual curation. The version of the genome that was deposited in TriTrypDB [36], and which was used for this analysis, includes 30 pseudochromosomes and the kinetoplast maxicircle.

### Data analysis

Sequenced reads from cultured adherent, cultured swimming, and infected *Ae*. *aegypti* hindguts were mapped to the *C*. *fasciculata* CfC1 transcriptome (version 28), while reads from control and infected hindguts were mapped to the *Ae*. *aegypti* transcriptome (Liverpool strain AGWG AaegL5.1) using Kallisto (version 0.43.0) [37]. Mapped reads were read into the R/Bioconductor environment using the Tximport package (version 1.0.2). For cultured *C*. *fasciculata* samples (swimming and adherent), between 32% and 35% of sequenced reads aligned to the *C*. *fasciculata* transcriptome. In infected hindguts, between 0.2% and 1% of reads mapped to the *C*. *fasciculata* transcriptome, while between 81% and 84% of reads from these samples mapped to the *Ae*. *aegypti* transcriptome. *Ae*. *aegypti* transcripts were summarized to genes. Data were filtered to remove genes expressed at low levels [counts per million (cpm) <1] and normalized with the calcNormFactors function from *limma* ([38], version 3.34.4) using the TMM method [38]. For analysis of cultured samples (swimming versus adherent, three replicates each), transcripts were filtered out if they had a cpm<1 in three of the six samples. For analysis that included *C*. *fasciculata* transcripts from infected mosquito hindguts, transcripts were filtered if they had cpm<1 in three of the cultured samples and in any of the infected hindgut samples. Hierarchical clustering using a maximum distance method and principal component analysis (PCA) were used to visualize the relationship between samples. To confirm that differences in read depth between samples generated from cultured cells and mosquito hindguts did not affect our differential gene analysis, we used sub-sampling to confirm our results. When one million sub-sampled reads from each sample were compared, over 80% of the differentially expressed genes identified in the full analysis were also identified in the sub-sampled analysis. *limma* was used to identify differentially regulated *C*. *fasciculata* transcripts across our three conditions (cultured adherent, cultured swimming, and mosquito hindgut, False Discovery Rate <0.01 and log_2_ Fold Change >1) [38]. For analysis of differentially regulated *Ae*. *aegypti* transcripts, the experimental design required three independent infections, which introduced a systemic “batch effect.” To address this, we introduced a batch correction using the SVA package ComBat function [39, 40]. This batch correction is generated using an empirical Bayesian framework which adjusts for known batch effects (e.g. separate cultures of parasites, generations of mosquitoes and infection levels). This adjusted data was then used for downstream differential gene expression analysis. *limma* was used to identify differentially expressed genes (False Discovery Rate <0.05 and log_2_ Fold Change >0.59) [38]. Graphics for RNAseq data were created using ggplot2 [41]. Analysis of predicted transcripts and gene ontology were performed on TriTrypDB [36] for *C*. *fasciculata* transcripts and VectorBase [42] and David [43, 44] for mosquito transcripts. Gene Set Enrichment Analysis (GSEA) was performed using version 3.0 of the software available from the Broad Institute [45, 46]. All 134 available Kyoto Encyclopedia of Genes and Genomes (KEGG) [47] pathways on TriTrypDB and two additional custom gene sets were analyzed for enrichment in cultured adherent and swimming *C*. *fasciculata* with the number of permutations set to 1000 and a permutation type of “gene_set”. The data discussed in this publication have been deposited in NCBI’s Gene Expression Omnibus [48] and are accessible through GEO Series accession number GSE132641 (https://www.ncbi.nlm.nih.gov/geo/query/acc.cgi?acc=GSE132641).

## Results

### *C*. *fasciculata* cultured adherent cells resemble parasites found in the insect host

**Fig 1:**
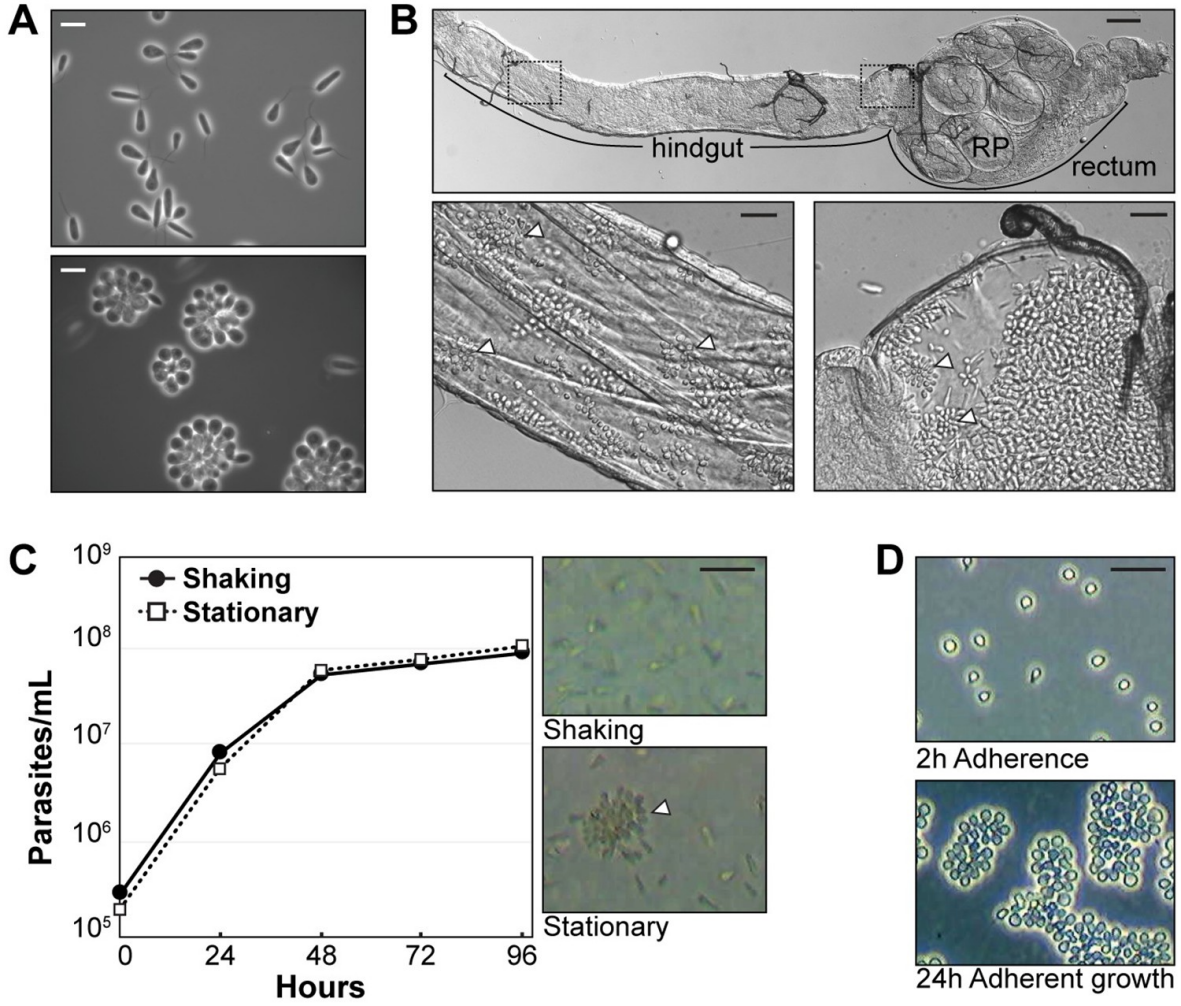
*Crithidia fasciculata* adherent stages in culture and the mosquito host. (A) Phase contrast images of live swimming (top panel) and adherent rosettes (bottom panel) grown in culture. Scale bars, 10 μm. (B) Images of *Crithidia* rosettes within an infected *Aedes aegypti* hindgut. Top panel shows an infected hindgut. *C*. *fasciculata* colonize the hindgut, rectum and rectal papillae (RP). Scale bar, 100 μm. Bottom panels are enlargements of the areas shown in the boxes above. Scale bars, 20 μm. Adherent cells are visible throughout the highlighted regions. White arrowheads in bottom panels indicate rosettes (C) Growth curve comparing rates of growth of cells grown in flasks kept on a rocker (shaking, solid line) and the incubator shelf (stationary, dotted line). Images of cells in shaking and stationary flasks. White arrowhead indicates a rosette. Scale bar, 25 μm. (D) Top image, cells that were allowed to adhere for 2 h followed by 3 washes to remove swimming cells. Bottom image shows the same flask after adherent cells have been allowed to grow for ~24 h. Scale bar, 25 μm.

It has been previously noted that certain kinetoplastid species can adhere to culture substrates, including glass and tissue culture plastic (Fig 1A, [24, 49, 50]). For *C*. *fasciculata*, adhesion in culture is favored by stationary incubation as opposed to incubation on a shaker or rocker. For this reason, researchers have typically grown their cultures with movement in order to maintain a relatively homogeneous population of swimming cells. *C*. *fasciculata* also adhere to the cuticular lining of the mosquito hindgut [19, 22, 51], presumably allowing effective colonization of this compartment.

We infected laboratory mosquito strains with *C*. *fasciculata* to confirm that adherent parasites found in mosquitoes morphologically resemble adherent forms grown in culture. Mosquitoes were allowed to feed *ad libitum* on sucrose containing flagellated swimming *C*. *fasciculata* for 24 hours, after which they were maintained on a standard sucrose diet. Seven days post-infection, mosquitoes were dissected and tissues examined for *C*. *fasciculata*.

Using this protocol, we achieved a nearly 100% infection rate for *Anopheles gambiae*, *Anopheles stephensi*, and *Aedes aegypti* mosquitoes. While small numbers of parasites were found in the crop and midgut, the vast majority of *C*. *fasciculata* cells were adhered to the lining of the hindgut and rectal papillae (Fig 1B). The parasites were found at high density, covering most of the surface of these organs. This indicates that the relatively small numbers of parasites reaching the mosquito hindgut were able to robustly colonize and replicate. The density of colonization and the percent of infected mosquitoes were comparable to what has been reported with *Culex* mosquitoes [52]. Despite high parasite numbers, under standard laboratory conditions we saw no difference in lifespan of infected mosquitoes (either males or females) when compared to uninfected control mosquitoes (S1 Fig).

Examination of *C*. *fasciculata* cells in infected hindguts and rectal papillae revealed that the parasites had transformed from the flagellated swimming (nectomonad) form used to feed the mosquitoes into adherent, sessile haptomonads. These cells are attached to the cuticular lining of these tissues and are often observed in rosette-like clusters similar to those observed in culture. In a freshly dissected sample, parasites are almost exclusively observed as haptomonads. However, after several minutes, motile nectomonads can be found and typically occur in damaged areas, perhaps due to prolonged incubation of the tissue in buffer, as observed previously [22]. As described below, we have observed swimming cells dividing from rosettes in culture; therefore, the presence of these motile cells could also be due to normal production of swimming cells during cell division within the growing rosettes.

### Stationary culture and removal of swimming cells promotes the transformation and growth of adherent *C*. *fasciculata*

We measured growth rates of *C*. *fasciculata* parasites cultured in stationary flasks compared to flasks that were agitated on a rocking platform (Fig 1C). Cells divided at similar rates; however, the rocking culture contained almost exclusively swimming nectomonad forms while the stationary flasks contained some adherent, haptomonad cells (Fig 1C). In order to study adherent *C*. *fasciculata*, we developed a cell culture protocol to enrich for adherent cells (Fig 1D). Cells from a mid-log phase culture were diluted into a new flask and incubated for 2 hours without agitation. We observed that a small number of single cells will adhere to the flask under these conditions. Non-adherent cells were removed by washing. Following 24 hours of stationary incubation, we found that individual adherent cells had divided to form large rosettes. We also observed a small number of swimming cells, which could be removed by washing, leaving a culture composed almost exclusively of adherent parasites. For our purposes we have defined a “rosette” as a group of cells that arose from the division of a single cell or pair of adherent cells, rather than a cluster of swimming cells that associate via their flagella, as has been observed for other organisms [2].

### Cell division within rosettes is rapid and produces swimming cells

**Fig 2:**
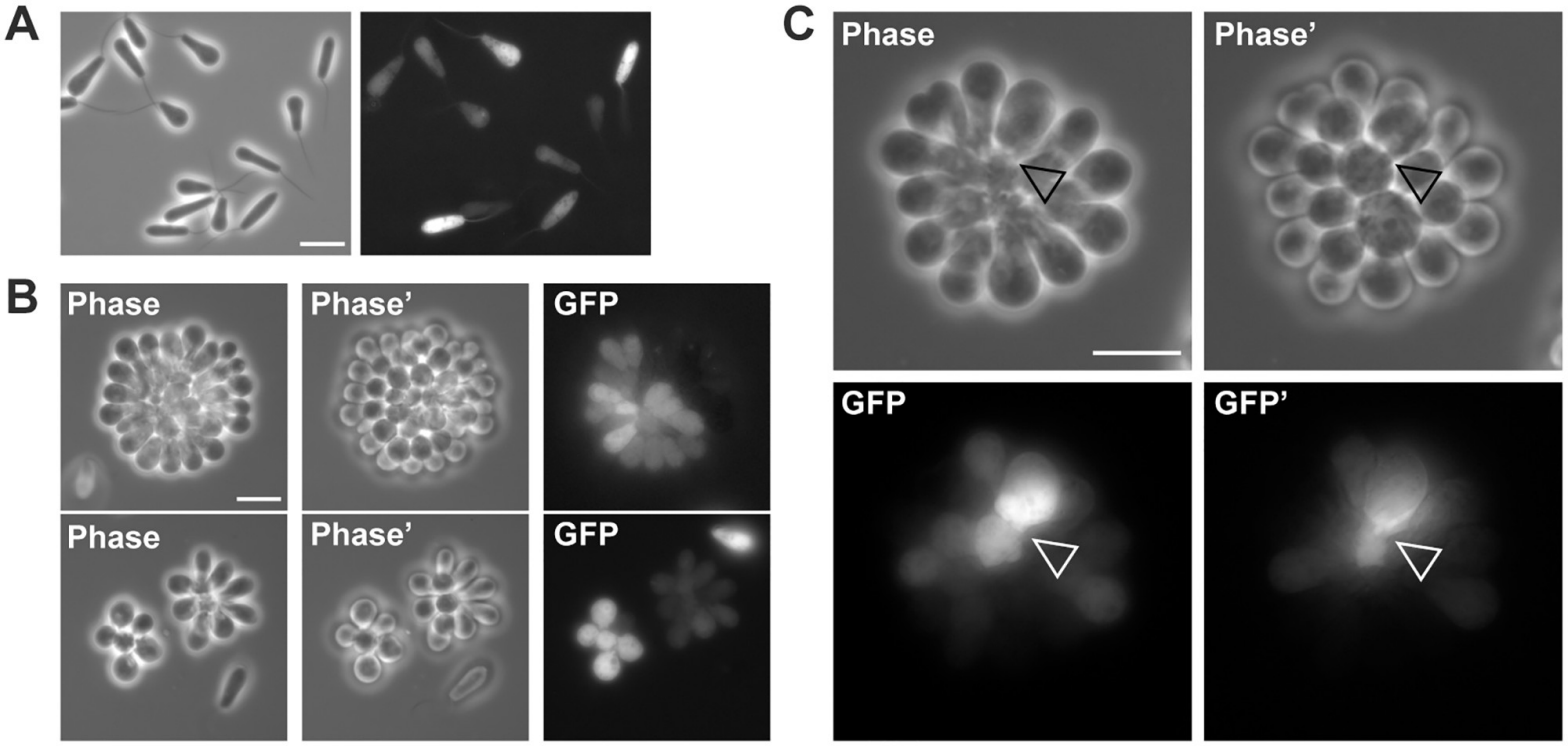
Organization of cultured *C*. *fasciculata* rosettes. (A) Live *C*. *fasciculata* swimming cells expressing cytoplasmic GFP from an episomal plasmid. Scale bar, 10 μm. (B) Live GFP-expressing rosettes grown on poly-L-lysine-coated glass coverslips. Two different focal planes are shown in phase contrast to highlight the three-dimensional structure of the rosette. Scale bar, 10 μm. (C) Enlargement of rosette cells expressing GFP. Arrowhead indicates possible junction between cells. Scale bar, 10 μm.

To visualize rosettes in more detail both in culture and in the mosquito, we created a *C*. *fasciculata* cell line expressing cytoplasmic GFP. In culture, this allowed us to visualize more clearly the relationships between individual cells in the rosette. Since our GFP plasmid is maintained episomally, some cells have reduced GFP expression due to imperfect inheritance of the plasmid (Fig 2A and 2B). Variability in GFP signal is most likely due to variable levels of expression in cells containing different numbers of episomal plasmids, as GFP signal did not correlate with either cell cycle or morphological form (swimming or adherent). The heterogeneity of GFP expression between cells in a rosette indicates that, despite their close apposition to each other, the cytoplasm of each cell remains separate (Fig 2B and 2C). However, we cannot rule out the presence of gap junction-like connections between these cells through which GFP is too large to pass.

**Fig 3:**
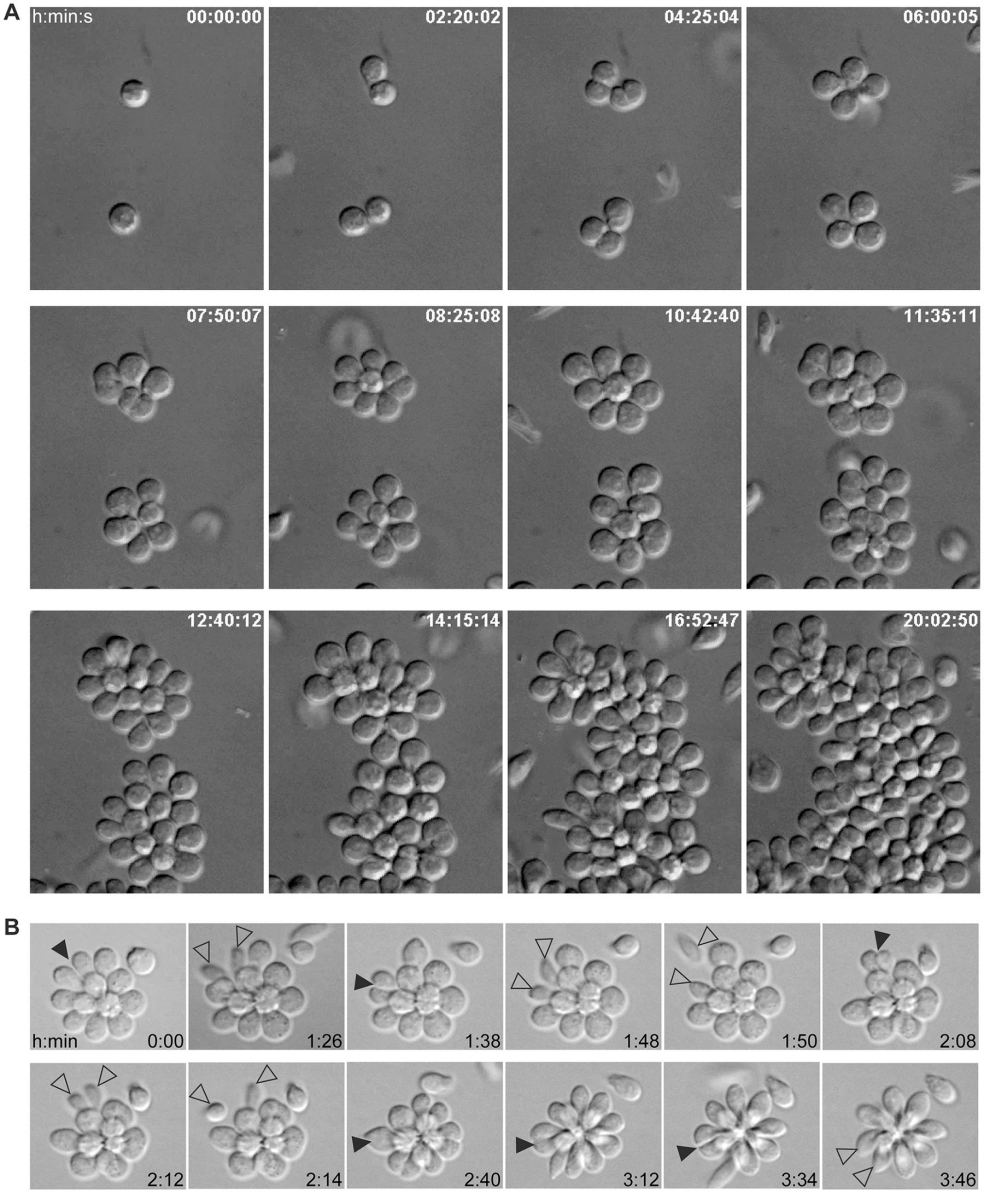
Time course Differential Interference Contrast live microscopy of cells in a rosette. (A) Panels are representative frames taken over 20 h of a 24 h imaging period. During this time, several cell divisions were observed. Times are shown as h:min:s. (B) Cells imaged live over approximately 4 h. Black arrowheads indicate cells in the process of division, while open arrowheads indicate divided cells about to leave the rosette. Times are shown as h:min.

To observe the behavior of adherent cells within a rosette, we used time-lapse video microscopy (Fig 3A; S1 and S2 Movies). Starting from single adherent cells, cell division in rosettes typically produces either two daughter cells that remain in the rosette as haptomonads, or two cells that initiate transformation into swimming nectomonads. When the latter occurs, the cell body of each daughter cell is more elongated post-division, presumably with growth of the flagellum, before both cells detach from the rosette almost simultaneously (Fig 3B; S3 Movie). Haptomonads continued to divide until they formed a near complete monolayer on the surface of the dish. Swimming nectomonads also accumulated during this time, likely due to their division from rosettes as well as their further division in the medium.

To quantitate the doubling time of haptomonads, we observed 92 cell divisions (46 cells) over a 24-hour period. The average time between cell divisions in rosettes was 3 hours 48 min (228 +/- 34 min). To compare this to swimming cells, we calculated swimming cell doubling time based on growth curves of nectomonad cultures grown on a rocker. When we did this for nine different time points in six separate cultures, we estimate an average doubling time of 4 hours 42 min (282 +/- 63 min). Although we used two different methods to estimate their doubling time, our results raise the possibility that adherent cells replicate more quickly than swimming cells.

### *C*. *fasciculata* is primarily observed adhering to the posterior gut of the mosquito

**Fig 4:**
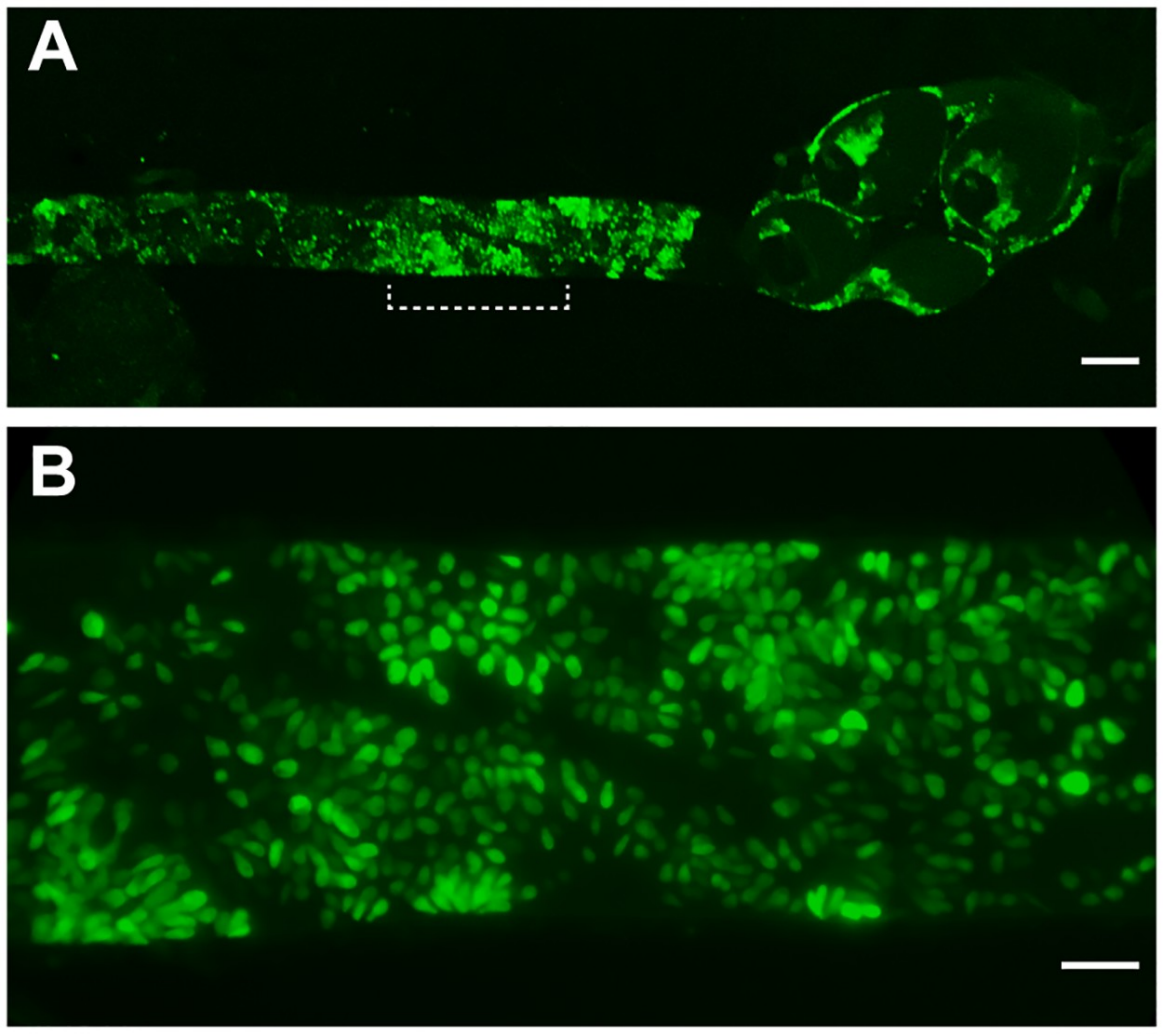
Hindgut of *Ae*. *aegypti* mosquito 7 days post infection with GFP-expressing *C*. *fasciculata*. (A) Widefield fluorescence microscopy was used to image fluorescent parasites in a dissected mosquito hindgut. Parasites are observed attached to the hindgut, rectum and rectal papillae. (B) Higher magnification image of the bracketed region indicated in panel A. The scale bars in panels A and B are 100 μm and 20 μm, respectively.

We took advantage of our GFP-expressing *C*. *fasciculata* cells to obtain clearer images of the parasites within mosquitoes. This approach highlighted the abundance and organization of the parasites along the lining of the hindgut, rectum and rectal papillae (Fig 4A). As described above, the majority of the cells are sessile haptomonad forms that grow in rosette-like patterns (Fig 4B). This approach also allowed us to examine other mosquito tissues for the presence of *C*. *fasciculata*. Despite their abundance in the hindgut and rectum, we were unable to detect large numbers of parasites in other organs of the mosquito, including the crop and the midgut. We suspect, in agreement with previous studies, that *C*. *fasciculata* is unable to adhere to or replicate within these tissues [19, 22].

### Transcriptomic analysis of swimming and adherent *C*. *fasciculata*

To uncover proteins involved in adherent growth, we performed transcriptomic analysis of adherent and swimming cells. We prepared RNA from three mid-log phase cultures of swimming and adherent *C*. *fasciculata* using the enrichment procedure described above. We also prepared RNA from dissected *Ae*. *aegypti* hindguts 7 days post-infection with *C*. *fasciculata* and from mock-infected controls. Bar-coded mRNA libraries were created for RNA sequencing analysis (RNAseq). In total, we analyzed 12 samples: 3 replicates each of cultured swimming, cultured adherent, uninfected female hindguts, and infected female hindguts.

**Fig 5:**
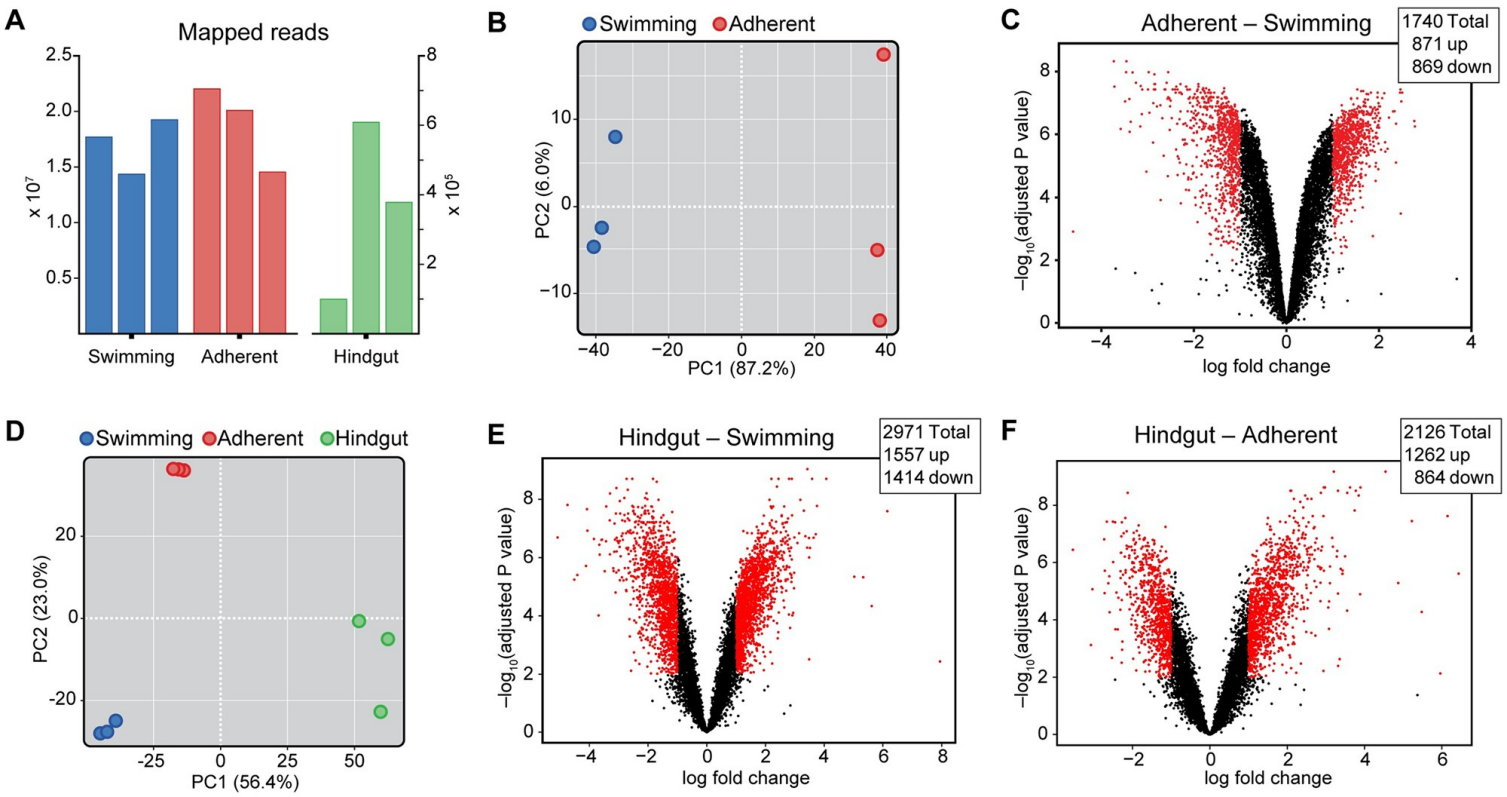
RNAseq analysis of swimming and adherent *C*. *fasciculata*. (A) The number of reads mapped to the *C*. *fasciculata* genome from cultured swimming, cultured adherent (left Y-axis), and infected mosquito hindgut samples (right Y-axis). Very few reads mapped in our mosquito samples, but subsampling indicates that our results are reproducible and not the result of differences in read depth. (B) Analysis of adherent and swimming *C*. *fasciculata* generated *in vitro*. Principle component analysis (PCA) shows a clear separation between cultured swimming and cultured adherent sequenced samples in PC1, indicating that adherence accounts for most of the variability between these samples. (C) Volcano plot showing transcript abundance in swimming vs. adherent cells (positive value indicates transcript is up in adherent cells). Dots shown in red represent transcripts with a log_2_ fold change ≥1 and an adjusted P value p<0.01. Numbers of differentially regulated genes are shown in the graph inset. (D) PCA of all samples including *C*. *fasciculata* reads from infected mosquito hindguts. (E) Volcano plot showing abundance of *C*. *fasciculata* transcripts in mosquito hindgut samples versus cultured swimming or (F) cultured adherent samples. Positive value indicates transcript is up in hindgut samples. Red dots represent transcripts with a log_2_ fold change ≥1 and an adjusted P value p<0.01. Numbers of differentially regulated genes are shown in graph insets.

We first mapped our sequenced reads to *C*. *fasciculata* Cf-C1 strain transcripts (obtained from TriTrypDB) using the Kallisto program [37]. Although we were able to map greater than 10^7^ reads for the cultured cell samples, this represented only ~30% of our total reads. This is likely due to incomplete transcript models given that ~80% of our reads align to the genomic DNA of the reference strain. Despite robust infection, the hindgut samples are dominated by mosquito transcripts with less than 1% of reads mapping to the *C*. *fasciculata* transcriptome (Fig 5A). In contrast, with more than 3 x10^7^ reads, the same sample had a mapping success rate of greater than 80% to *Ae*. *aegypti* transcripts, similar to the uninfected hindgut samples (S2 Fig). Based on the number of *C*. *fasciculata* mapped reads, we conclude that replicate two had the highest infection levels, followed by replicate three and replicate one (Fig 5A). Because of the small number of mapped *C*. *fasciculata* reads in the mosquito samples, our initial analyses dealt only with cultured cells.

Principal component analysis (PCA) of the two cultured samples showed a clear separation between swimming and adherent cells along PC1, which accounted for 87.2% of the variation between samples (Fig 5B). We then compared adherent to swimming samples to identify differentially regulated transcripts. We found 1740 transcripts that were differentially regulated by at least 2-fold, of which 871 were upregulated in adherent cells relative to swimming cells, while 869 were downregulated (Fig 5C; S1 and S2 Tables). We verified 3 of the most strongly up- and down-regulated single-copy genes by qRT-PCR in an independent set of samples (S3 Fig).

We found much greater variability in our mosquito samples than those derived from cultured parasites. This is likely because the three replicates of infected mosquito hindguts were derived from separate generations of mosquitoes and separate cultures of *C*. *fasciculata*. Because we obtained fewer *C*. *fasciculata* reads from these samples, we applied more stringent filtering conditions to eliminate low expression transcripts. Transcripts were eliminated if the cpm was <1 in three of the cultured samples or any of the mosquito samples. PCA of all nine samples under these conditions showed that, while cultured *C*. *fasciculata* of either form differed from parasites in the hindgut, PC1, which accounts for 56.4% of the variation between the samples, suggests the hindgut-adhered cells are more closely related to the adherent form (Fig 5D). Consistent with cultured/not-cultured being a major determinant for variability, we also found a higher number of differentially expressed genes when *C*. *fasciculata* samples from the mosquito hindgut were compared to either cultured swimming (Fig 5E) or cultured adherent parasites (Fig 5F), than when the cultured samples were compared to each other.

**Fig 6:**
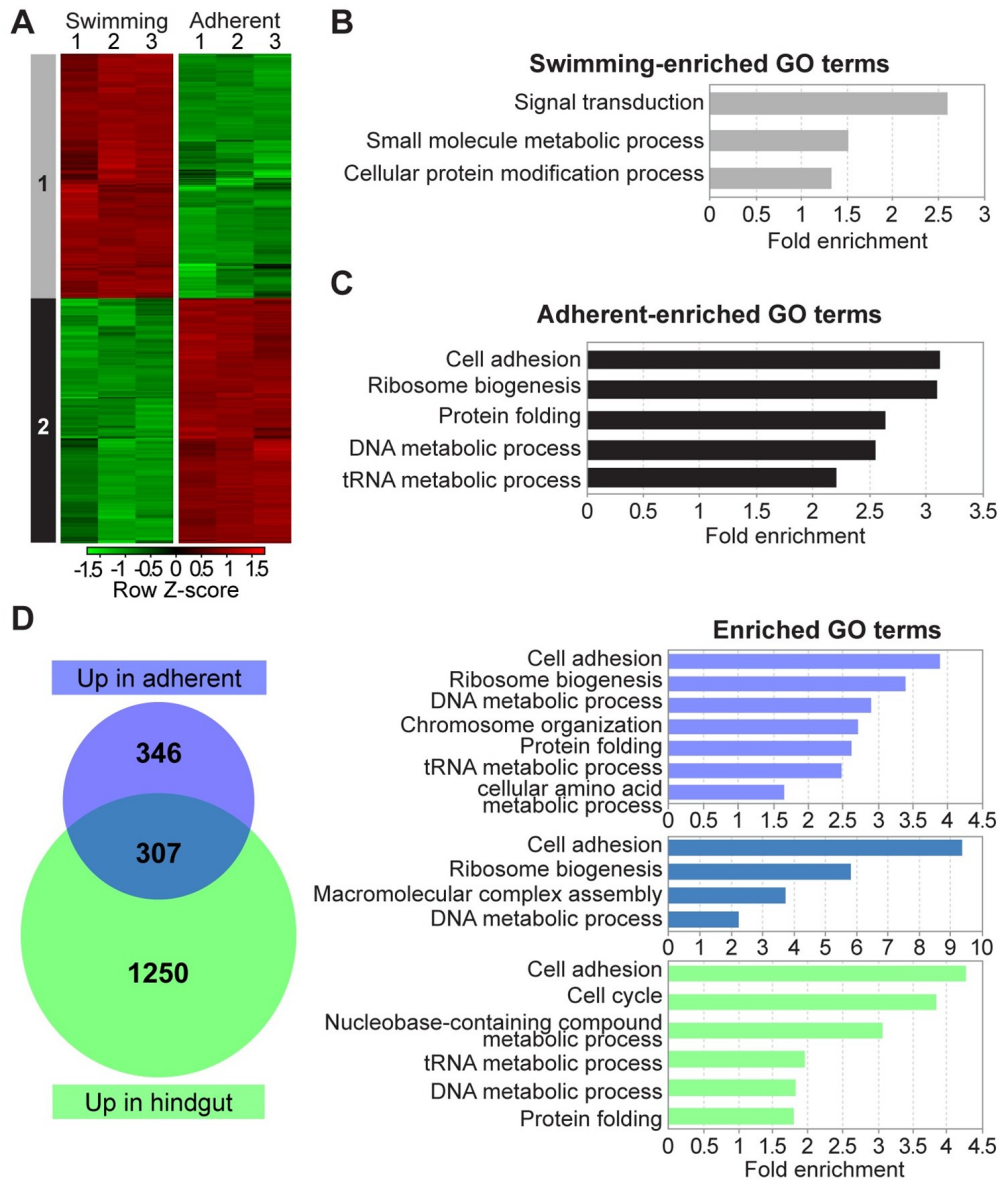
Differentially expressed genes in cultured swimming and cultured adherent *C*. *fasciculata*. (A) Heat-map showing gene expression levels of genes differentially expressed by at least two-fold with an adjusted P value p<0.01 across three replicates of swimming cells and three replicates of adherent cells. Replicate number is shown at the top of the heat map. Each row represents a gene, and rows were clustered according to their expression pattern. Group 1 genes are upregulated in swimming cells relative to adherent cells. Group 2 genes are downregulated in swimming cells relative to adherent cells. Red, upregulated; green, downregulated. (B)(C) Gene ontology (GO) enrichment analysis (using GO Slim terms) of genes in groups shown in A was performed on TriTrypDB. p<0.05. (D) Analysis of differentially expressed genes in all nine samples (three replicates each of swimming, adherent, and infected hindguts). Venn diagram showing the degree of overlap in transcripts upregulated in cultured adherent parasites and hindgut-adherent parasites (both relative to cultured swimming cells). GO Slim terms enriched in each group (p<0.05) are shown in color-coded graphs on the right.

For our analysis of adherence *in vitro*, we clustered differentially regulated genes according to expression across our cultured cell replicates (Fig 6A) and identified two main groups, those upregulated in swimming cells relative to adherent cells (Group 1), and those upregulated in adherent cells relative to swimming cells (Group 2). We then examined these clusters by gene ontology (GO) analysis, limiting our enrichment analysis to a subset of GO Slim terms. The group of transcripts upregulated in swimming cells was enriched for genes with predicted roles in signal transduction, small molecule processes, and protein modification (Fig 6B). A closer look at the signal transduction and small molecule categories revealed a number of genes implicated in cyclic AMP signaling, including nine predicted receptor-type adenylate cyclase genes, an oxygen-sensing adenylate cyclase, and three cyclic nucleotide phosphodiesterases. In addition, this group included three phosphoinositide phospholipases. Of the 40 genes with the GO Slim term for protein modification, 36 are kinases. Intriguingly, the predicted repressor of differentiation kinases 1 and 2, which are involved in developmental transitions in *T*. *brucei*, are both in this list [53].

We then performed a similar GO analysis on genes that are upregulated in adherent relative to swimming cells. There were five GO Slim categories that were significantly enriched: cell adhesion, tRNA metabolism, ribosome biogenesis, protein folding, and DNA metabolism. All five of the cell adhesion genes are annotated as *gp63*/leishmanolysin and are located in the same region of chromosome 4. The GP63 proteins have been implicated in cell adhesion in *Leishmania* species [14, 54] as well as other kinetoplastids [13, 15, 16, 55], and will be discussed in more detail in a later section.

Since the PCA of all nine samples across three conditions indicated that the transcriptome of adherent cultured cells rather than that of swimming cells more closely resembles cells adhered to the mosquito hindgut, we compared the list of genes upregulated in each adherent cell type (cultured and in the hindgut) relative to cultured swimming cells (Fig 6D). 1557 transcripts were found to be upregulated in hindgut-adhered compared to cultured swimming cells (S3 Table). Enriched GO terms for this group include cell adhesion, cell cycle, nucleotide metabolism, tRNA metabolism, DNA metabolism, and protein folding. In this analysis, which used a filter to remove genes that did not have data in all three hindgut samples, 653 transcripts were upregulated in cultured adherent cells relative to swimming cells. Of these, approximately half (307) were also upregulated in hindgut-adhered cells (S4 Table). This includes the five *gp63* genes as well as genes with GO Slim terms for adhesion, DNA metabolism, macromolecular complex assembly, and ribosome biogenesis. In contrast, of the 773 genes upregulated in swimming cells, only 196 were also upregulated in hindgut-adhered cells (relative to cultured adherent cells), and for this group no GO Slim terms were significantly enriched.

**Fig 7:**
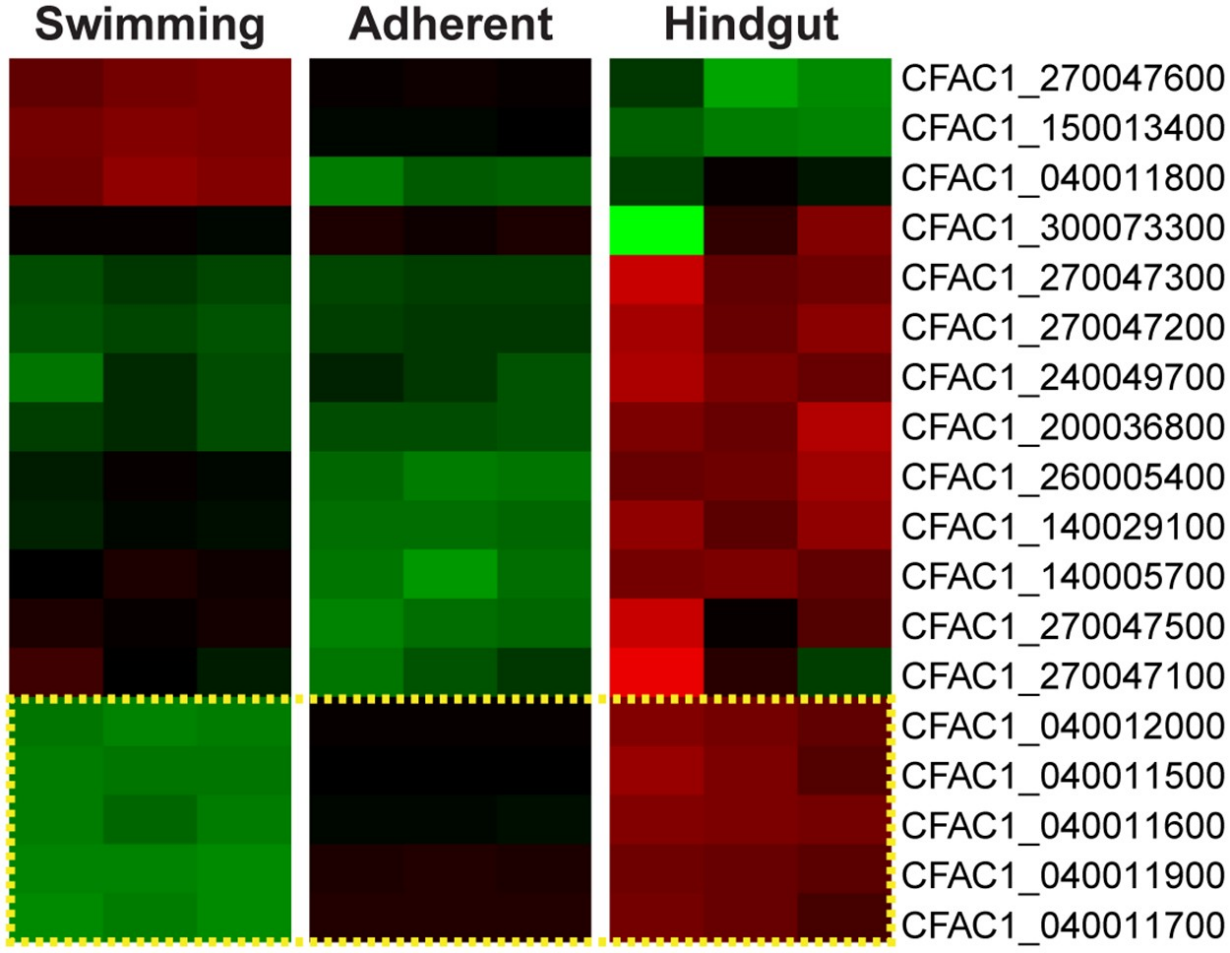
*gp63* genes are differentially expressed in adherent versus swimming *C*. *fasciculata*. Heat map showing gene expression patterns of predicted *gp63* genes in the *C*. *fasciculata* Cf-C1 genome across our different samples. The five genes outlined by the yellow box were significantly enriched in our cultured adherent samples by at least two-fold with an adjusted P value p<0.01.

Since we found a number of predicted *gp63* genes in our lists of differentially regulated transcripts, we examined the expression pattern of all 18 predicted *gp63* and *gp63*-like genes in the *C*. *fasciculata* genome across our nine samples. As mentioned above, in addition to the five *gp63* genes that are upregulated in both cultured adherent and mosquito-stage *C*. *fasciculata* (Fig 7, yellow box), five more *gp63* genes were significantly upregulated in mosquito-stage parasites that were not identified as differentially regulated in adherent cultured cells (Fig 7). Interestingly, three *gp63* genes were significantly upregulated in swimming *C*. *fasciculata* relative to cultured adherent cells. Two of these were also significantly higher in swimming cells relative to hindgut-adhered cells. To confirm these patterns of regulation, we analyzed seven *gp63* genes by qRT-PCR in independent samples from cultured adherent and swimming *C*. *fasciculata* (S4 Fig). As expected, three of these genes were upregulated in swimming cells and three were upregulated in adherent cells. In addition, we found that the hindgut-specific *gp63* was also upregulated in our cultured adherent cells. These data confirm the differential regulation of a subset of *gp63* genes identified by RNAseq and indicate that members of this family may be important in the different developmental forms of *C*. *fasciculata*.

### Pathway analysis

**Fig 8:**
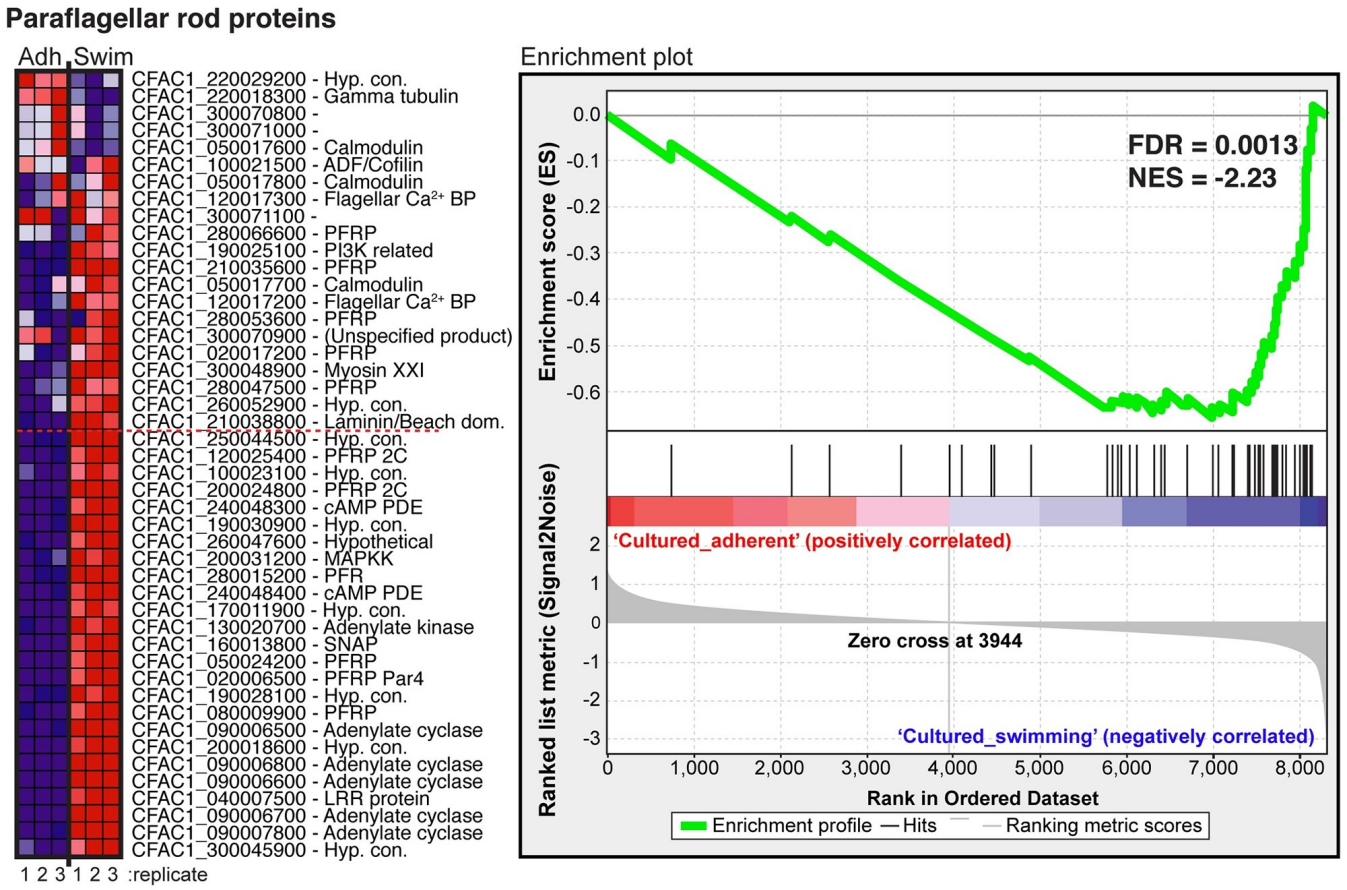
GSEA of putative paraflagellar rod proteins. The left panel shows a heat map of enrichment in adherent (Adh) or swimming (Swim) forms for the three individual replicates (bottom of image). Gene identifiers and TriTrypDB gene name are shown. Red color indicates transcript enrichment in swimming form and blue indicates enrichment in adherent form. The 25 genes below the red dashed line (GSEA software threshold) are considered as significantly contributing to the enrichment of the set. The right panel shows the enrichment plot generated by the GSEA software with the Normalized Enrichment Score (NES) and False Discovery Rate (FDR) indicated for this gene set. A negative NES indicates enrichment in swimmers. The gray shaded area shows a rank order of the genes based on their enrichment in adherent (left, red) or swimming forms (right, blue). The black vertical lines indicate the position of the 46 members of the set within the rank order. The density of lines on the right indicates the strong enrichment of genes in swimming form. The green line indicates the enrichment profile generated by the software.

To further functionally analyze differentially regulated genes in adherent and swimming cells, we performed Gene Set Enrichment Analysis (GSEA) [45, 46]. Our analysis was focused primarily on predicted metabolic pathways. Of the 134 KEGG pathways available from TriTrypDB, 94 had greater than 14 member genes present in the dataset and were included in our analysis, while 40 were rejected based on this criterion (S5 Table). We also included a gene list of all predicted GP63s. Finally, given that several transcripts of genes with a putative function in the paraflagellar rod were upregulated in swimming cells, we also included a set of genes encoding predicted *Crithidia* paraflagellar rod (PFR) proteins [56]. GSEA analysis revealed 13 gene lists enriched in adherent form and 10 for swimming forms (Table 1). Interestingly, of all 96 gene sets, putative paraflagellar rod genes had the strongest enrichment score of all the sets analyzed with 54% (25 out of 46) of the members enriched in swimming cells with a false discovery rate less than 0.001 (Fig 8). This is consistent with the observation that swimming cells possess a prominent flagellum and are highly motile. The strong enrichment of paraflagellar rod transcripts in swimming cells indicates that differences in gene regulation likely reflect functional differences between different cell types and suggests that GSEA is a robust tool for analyzing our data and generating new hypotheses. The *gp63* gene set was not significantly enriched in either form. This was expected given that, within this family, different genes were enriched in both swimming and adherent forms (see Fig 7).

**Table 1:** Gene sets enriched in adherent and swimming form *C*. *fasciculata*.

**Enriched in adherent**				
*NAME*	*KEGG*	*Size*	*NES*	*FDR*
Seleno compound metabolism	ec00450	51	2.057	0.000
Biosynthesis of ansamycins	ec01051	55	1.914	0.003
Flavonoid biosynthesis	ec00941	55	1.910	0.002
Aminoacyl-tRNA biosynthesis	ec00970	31	1.910	0.002
Porphyrin and chlorophyll metabolism	ec00860	80	1.903	0.002
Biosynthesis of 12-14-and16-membered macrolides	ec00522	56	1.902	0.001
Polycyclic aromatic hydrocarbon degradation	ec00624	55	1.809	0.006
Stilbenoid diaryl heptanoid and gingerol biosynthesis	ec00945	58	1.804	0.005
Anthocyanin biosynthesis	ec00942	71	1.736	0.012
Ubiquinone and other terpenoid-quinone biosynthesis	ec00130	79	1.727	0.012
Histidine metabolism	ec00340	68	1.717	0.012
Insect hormone biosynthesis	ec00981	87	1.669	0.019
Cysteine and methionine metabolism	ec00270	96	1.580	0.037
**Enriched in swimmers**				
*NAME*	*KEGG*	*Size*	*NES*	*FDR*
Putative paraflagellar rod proteins	NA	46	-2.230	0.001
Valine leucine and isoleucine degradation	ec00280	33	-2.090	0.001
Fatty acid degradation	ec00071	36	-2.080	0.001
Caprolactam degradation	ec00930	15	-1.950	0.003
Starch and sucrose metabolism	ec00500	28	-1.920	0.004
Oxidative phosphorylation	ec00190	55	-1.840	0.01
Fatty acid elongation	ec00062	20	-1.820	0.012
Glycerolipid metabolism	ec00561	39	-1.760	0.02
Propanoate metabolism	ec00640	50	-1.720	0.026
Biosynthesis of unsaturated fatty acids	ec01040	22	-1.710	0.028

The pathway or gene set name, KEGG pathway ID (where applicable), number of genes (Size), GSEA Normalized Enrichment Score (NES), and False Discovery Rate q-value (FDR) are provided. We considered FDR < 0.05 as enriched. Positive and negative NES scores indicate gene set enrichment in adherent or swimming form cells, respectively.

Besides PFR genes, gene sets for fatty acid metabolism and oxidative phosphorylation were also upregulated in swimming cells (Table 1). In adherent cells, a gene set of aminoacyl-tRNA synthetases was enriched, including 18 predicted synthetases (all except those for alanine and tryptophan) (S5 Fig). The remaining adherent-enriched gene sets consisted mainly of the same core group of 24 genes, many of which have predicted roles in rRNA and tRNA metabolism. Outside of this core group, some genes specific to individual gene sets were also enriched, including seven genes with predicted roles in insect hormone biosynthesis and seven with predicted roles in cysteine/methionine metabolism (S5 Table).

### Mosquito cell signaling and immune genes respond to infection by *C*. *fasciculata*

**Fig 9:**
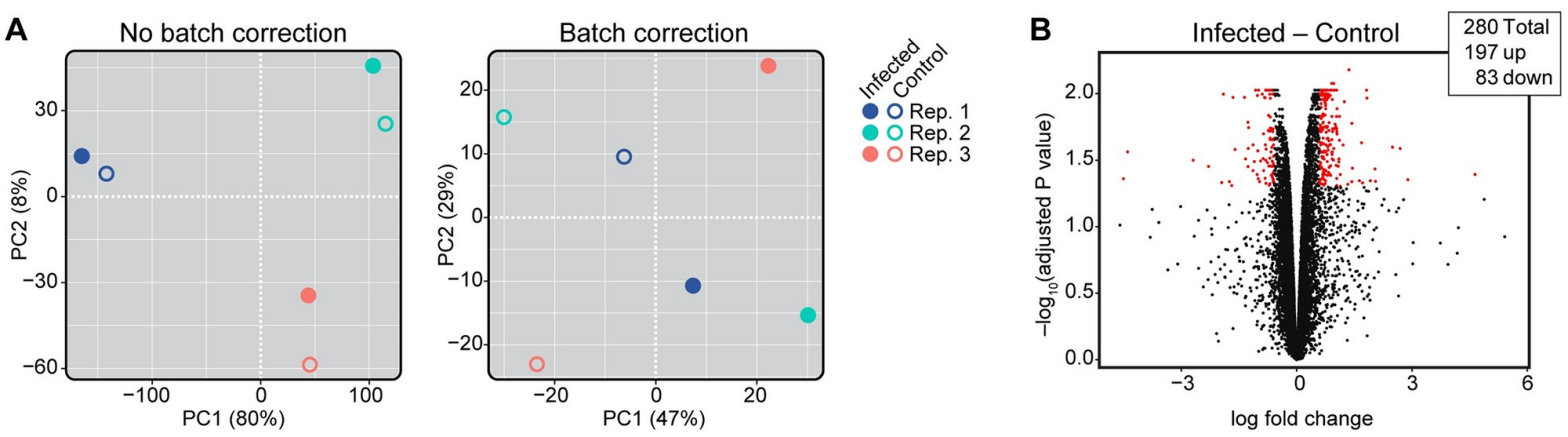
Differentially regulated genes in *C*. *fasciculata*-infected *Ae*. *aegypti* hindguts. (A) PCA analysis shows that before batch correction (left) the most significant source of variation is experimental replicate (Rep; different colors). Applying batch correction (right) separates control, uninfected (open circles) from infected samples (solid circles) along PC1, which now accounts for 47% of the variation between samples. (B) Volcano plot showing changes in gene expression in infected hindguts compared to uninfected hindguts. A positive value indicates an *Ae*. *aegypti* transcript that is upregulated in infected hindguts. Red dots indicate transcripts that are significantly differentially expressed. Numbers of differentially expressed genes with a log_2_ fold change of at least 0.59 and adjusted P value < 0.05 are shown in the inset.

Since the mosquitoes exposed to *C*. *fasciculata* were robustly infected, we asked whether there was a transcriptional response in the affected tissue, the hindgut. To do this we mapped our reads from uninfected and infected mosquitoes onto the *Ae*. *aegypti* transcriptome (S2 Fig) and assayed for infection-induced changes in gene expression. Our PCA analysis revealed that the individual replicates were a significant source of variability comprising the first two principle components, PC1 and PC2, and accounting for approximately 88% of the differences (Fig 9A). Principle component 3 accounted for about 5% of the variation and was due to differences in infection levels between replicates. Therefore, most of the variation likely arises from the fact that separate generations of mosquitoes and independent cultures of *C*. *fasciculata* were used for each replicate. To account for these differences, we re-analyzed the samples using a batch correction method that is better suited to identify consistent differences between infected and uninfected hindguts in each replicate. Indeed, using batch correction, infection now determines PC1 and accounts for 47% of the variation between samples (Fig 9A). This analysis revealed 280 differentially regulated genes, 197 of which were upregulated in infected tissues and 83 of which were downregulated (Fig 9B and S6 and S7 Tables).

Within the set of differentially regulated genes are 12 immune proteins. Of the 5 upregulated, three are antimicrobial peptides (AMPs), DEFA, DEFC, and DEFD. The other two upregulated genes are the Peptidoglycan recognition protein PGRPLB and a C-type lectin. Interestingly, 7 additional immune genes were downregulated including 4 putative pattern recognition molecules LRIM10B, LRIM7, CTL20, and CTLMA12. Three other putative immune genes were downregulated in *C*. *fasciculata*-infected hindguts. The most significantly downregulated gene is a Reeler domain-containing protein with a putative role in the extracellular space and which has been shown in silkworm larvae to play a role in nodule formation and melanization during bacterial infection [57]. The final two downregulated immune genes are a CLIP domain serine protease and a member of the Spätzle family. In addition to immune signaling, Spätzle proteins may also function in development and differentiation. One possibility is that *C*. *fasciculata* infection causes increased enterocyte cell death, requiring differentiation of intestinal stem cells in order to replace them. In support of this, genes with a predicted function in cell signaling are abundant in the list of differentially regulated genes. Of the 34 putative signaling genes, 31 are upregulated including those with predicted functions in Ecdysone, Insulin, Wnt, Hippo, and MAPK pathways.

## Discussion

Pathogenic kinetoplastid parasites are responsible for significant human disease burden worldwide. Although they have diverse life cycles, almost all kinetoplastids share similarities at both the morphological and genomic levels. One of the core features shared by these parasites is the ability to adhere to tissues in their insect hosts. This property is also found in non-pathogenic species such as *C*. *fasciculata*. We are exploring the possibility that *C*. *fasciculata*, which adopts an adherent form both *in vitro* and *in vivo*, is a viable model for studying the process of kinetoplastid adhesion. We have shown that the transformation from swimming to adherent cells results in substantial changes in gene expression, most likely at the level of transcript stability since kinetoplastids as a group have very little transcriptional regulation [58, 59]. The number of genes differentially regulated, even in cultured cells where both forms have the same nutritional environment, supports the idea that adherent cells are indeed a distinct developmental stage.

Our current understanding of attachment is that it is initiated via the flagellum or flagellar pocket region, as has been shown for other kinetoplastids [3, 49, 60–63]. This attachment presumably signals a developmental program that leads to dramatic shortening of the flagellum, formation of the adhesive structure, and accompanying changes in gene expression. The adhered cell can then replicate as the adherent form, producing a cell cluster called a rosette.

The ability of *C*. *fasciculata* to adhere to artificial substrates suggests that adherence may not require a specific receptor-ligand interaction. In other kinetoplastids, carbohydrates have been shown to play a role in adhesion. Lipophosphoglycan (LPG), the major surface glycoconjugate of *Leishmania* promastigotes, has been shown to mediate parasite adhesion to the sandfly midgut via an insect galectin [3, 54]. Adherence of *T*. *cruzi* to the midgut of *R*. *prolixus* can be blocked by incubation with carbohydrates or with lectins [64], and a similar effect was observed in the interaction between *T*. *rangeli* and *R*. *prolixus* salivary glands [65]. In *C*. *fasciculata*, it has been shown that a subpopulation of stationary phase cells fails to agglutinate in the presence of peanut lectin, an indication of changes in the composition of surface carbohydrates [66]. In *L*. *major* and *L*. *infantum*, this change is a marker for differentiation to the infectious metacyclic stage [67, 68]. Therefore, the finding that a small number of stationary phase *C*. *fasciculata* cells fails to agglutinate with peanut lectin could mean that this subpopulation is primed for differentiation, and that changes in surface carbohydrates are part of this transition [66].

Following the initial stages of kinetoplastid attachment, a distinct adhesive structure forms that is reminiscent of a hemidesmosome. Hemidesmosomes and desmosomes of higher eukaryotes are composed of intermediate filaments, the existence of which has never been conclusively shown in a unicellular eukaryote, although similar proteins have been identified in apicomplexans [69]. Intriguingly, bundles of filaments of unknown composition, but of a similar diameter to intermediate filaments, have been observed in *Phytomonas* and *C*. *fasciculata* [70, 71]. It is possible that these filaments are part of the kinetoplastid hemidesmosome and are composed of novel proteins with a structure similar to that of intermediate filaments. In addition, there is evidence that a protein or proteins of molecular weight ~70 kDa is involved in adherence of *T*. *congolense* to artificial substrates. A protein of similar size was identified as a putative component of novel filament structures in *C*. *fasciculata* [60, 70]. Of the 871 genes that are enriched in adherent cells, 28 have a predicted molecular weight between 68 and 72 kDa, including two predicted GP63s and 10 hypothetical conserved proteins. While these are candidates for future functional analysis, proteomic studies may also provide insights into the adhesive structures formed by these parasites.

Several predicted *gp63* transcripts were enriched in adherent cells both from culture and from the hindgut. GP63s, also known as Leishmanolysins, are GPI-anchored surface metalloproteases that are involved in host-parasite interactions and adhesion in other kinetoplastid species, particularly *Leishmania* (reviewed in [72, 73]). These proteins were initially described as mediating evasion of attack by vertebrate complement as well as attachment to and invasion of mammalian host cells by *Leishmania* parasites [72, 74, 75]. They were later proposed to mediate interactions between certain *Leishmania* species and their insect hosts [14]. GP63 proteins are found in all kinetoplastid species examined to date, including plant pathogens like *Phytomonas* [13] and monoxenous parasites of insects such as *C*. *fasciculata* [76]. The function of GP63 proteins in monoxenous parasite species, such as *C*. *deanei*, *C*. *guilhermei* and *Herpetomonas megaseliae*, has been investigated, and was consistent with a role in adhesion to insect tissues [16, 55]. In fact, it has been suggested that the ancestral role of GP63 proteins was to mediate adhesion of parasites to tissues in the insect, and that expansion of this protein family in the *Leishmania* lineage allowed interaction with mammalian macrophages [15, 55, 77]. The exact role of GP63s in adhesion is unclear. Since they are proteases, they could help to remodel substrates or tissues in order to facilitate adherence. GP63s may also play a nutritional role, allowing digestion of proteins for use by the parasites [78]. In addition to *gp63*s, 12 other predicted proteases or peptidases were enriched in adherent form *C*. *fasciculata*, two of which have predicted signal peptides and multiple transmembrane domains. In mosquito-stage parasites, 29 proteases/peptidases were upregulated, six of which were also upregulated in cultured adherent cells. These also may play a role in modifying the host tissue to facilitate growth in the mosquito hindgut.

It is interesting that we found certain *gp63* transcripts enriched in adherent cells, while others were enriched in swimming cells. It is possible that different subsets of GP63 proteins are associated with different forms of the parasite, allowing survival in different environments. Consistent with this, some GP63 proteins are differentially expressed during the life cycle of *Leishmania* [79]. Alternatively, the different groups of GP63 proteins might mediate different phases of the adhesion process, whereby those that are abundant in swimming cells are involved in initial adhesion while those expressed later help form a more durable adhesive structure.

In addition to structural components, we also expect that signaling molecules will act to initiate and maintain the developmental program resulting in an adherent cell. Transcripts with predicted roles in the cAMP signaling pathway were upregulated in swimming nectomonads, implying that cAMP signaling could maintain cells in the swimming state. Alternatively, this pathway could be important for registering the initial attachment event that triggers differentiation to haptomonads. In this model, differentiation to the adherent form leads to a downregulation of cAMP pathway components. cAMP signaling, specifically in the flagellum, has been implicated in social motility in *T*. *brucei* [80, 81]. Social motility is the coordinated behavior of populations of parasites. It is induced by surfaces and can act in trans for communication between cells. While social motility has not yet been demonstrated in *C*. *fasciculata*, our findings raise the possibility that cAMP signaling may be important for insect-specific behaviors, including regulation of adhesion between parasites as well as adhesion of parasites to the insect tissue.

In swimming cells, several genes involved in mitochondrial oxidative phosphorylation are upregulated, perhaps due to the amount of ATP required for motility. This suggests that mitochondrial function may be developmentally regulated in *C*. *fasciculata* as it is in other parasites such as *T*. *brucei* [82]. In the list of genes enriched in both cultured adherent and hindgut-adhered cells, we also found a number of genes with predicted functions in nucleic acid metabolism. These include genes that may be involved in transcription, chromatin structure, and RNA stability, perhaps contributing to the dramatically different transcriptomic profile observed in adherent cells. Interestingly, genes for both nuclear and mitochondrial DNA replication were also upregulated in adherent cells, along with components of the translation machinery. Upregulation of these proteins may allow for rapid proliferation during colonization of the mosquito. Our measurement of cell doubling times supports a faster replication rate in haptomonads. It’s possible that *C*. *fasciculata* in the natural environment (swimming nectomonads) divide more slowly to conserve resources, awaiting uptake by the mosquito host. Nevertheless, since we also observe an increase in these transcripts in adherent cultured cells, we suspect that this phenomenon is part of the developmental program initiated by attachment rather than a response to nutrient availability.

While we found a large number of differentially regulated genes when we compared adherent to swimming *C*. *fasciculata* found in culture, we found even larger differences when either of these samples were compared to *C*. *fasciculata* transcripts detected in infected mosquitoes. These differences could reflect an adaptation by the parasites to the environment of the mosquito hindgut relative to the culture environment. However, the similarities we were able to observe between adherent cells in culture and parasites in the mosquito support the utility of the culture model for dissecting the essential nature of the adhesive structure itself as well as the signaling components involved in developmental transitions between the two stages. To address aspects of mosquito-specific *C*. *fasciculata* biology, the robustness of our infection model should facilitate functional analysis in the insect host.

Using dual RNAseq, we have also provided evidence that the mosquito hindgut responds transcriptionally to the presence of *C*. *fasciculata* parasites. Although this may not seem surprising due to the high level of infection seen in our dissected mosquitoes, it is an open question as to how insect vectors sense and respond to microbes that occupy the hindgut. In particular, most of the work on mosquito innate immunity has focused on reactions occurring in the hemolymph. Kinetoplastid parasites usually remain in the gut or the salivary glands of their insect hosts and therefore do not encounter the hemolymph. One possibility is that *C*. *fasciculata* compromises the integrity of the hindgut lining, allowing bacteria to interact with the epithelium, provoking an immune response leading to AMP upregulation as well as increased epithelial cell turnover. If this is indeed the case, it highlights the robust nature of the mosquito’s innate immune system and developmental plasticity since mosquito survival is not changed by *C*. *fasciculata* infection (S1 Fig). The presence of parasites in the hindgut also seems to reduce the abundance of certain immune genes, perhaps allowing immune tolerance and successful colonization. It will be interesting to determine how this immune modulation affects the mosquito’s ability to transmit other pathogens, such as *Plasmodium*, viruses, and filarial nematodes. Elucidating mosquito responses to *C*. *fasciculata* in the hindgut may shed light on how other vectors respond to the presence of kinetoplastids in the gut. Further, it is not known how the presence of these parasites impacts the mosquito microbiome, which could also affect immune responses as well as the mosquito’s capacity to transmit other pathogens. For example, it is intriguing to speculate that the Reeler domain protein, with a putative role in melanization, may be involved in protecting the hindgut from bacterial commensals and also interferes with *C*. *fasciculata* adherence. Thus, when this gene is downregulated during infection, *C*. *fasciculata* are better able to colonize the hindgut, but bacteria can also come into contact with the epithelium leading to activation of AMPs.

Antimicrobial peptides were also upregulated in another model for kinetoplastid-insect interaction, infection of *Drosophila* by *Herpetomonas muscarum* [83]. In this system, transcriptional activation of AMPs was mediated by NF-κB signaling, which has similarly been shown to regulate Defensins A, C, and D in *Aedes aegypti* [84]. Although *Drosophila* are not a natural host of *C*. *fasciculata*, these parasites will colonize the gut following oral infection, triggering increased AMP production by the fat body. These findings all suggest that kinetoplastids in the gut can provoke a response in their insect host, one that may be relevant to transmission of pathogenic species. While the details of the interaction between parasite and insect may differ, particularly for parasites like *T*. *brucei* and *Leishmania* that primarily reside in the midgut, there is probably also a great deal of shared biology that may be revealed through study of the *C*. *fasciculata*-*Ae*. *aeypti* infection model. In addition, this model may be particularly relevant for *T*. *cruzi* which, like *C*. *fasciculata*, colonizes the hindgut and rectum of its insect vector.

We have shown that *C*. *fasciculata*, an important model for kinetoplastid biology, may also serve as a model for the conserved process of kinetoplastid adhesion to insect tissue. With a sequenced genome and the development of high-throughput genetic methods, like the recently described CRISPR/Cas9 system in *Leishmania* [85], a large number of gene candidates can be functionally screened using an *in vitro* adhesion assay. We are now using this method to try to generate knockout lines deficient in adhesion which can then be tested in our mosquito infection model to evaluate their role *in vivo*. This current work as well as future studies in *C*. *fasciculata* may provide candidates for targeted investigation of orthologous proteins in other insect-kinetoplastid interactions.

## Supporting information

S1 Fig *C*. *fasciculata* infection does not alter fitness of mosquitoes. Kaplan-Meier survival plots of *Ae*. *aegypti* following overnight feeding with *C*. *fasciculata* in a sucrose meal. Under these conditions virtually all mosquitoes are infected. (A) Male and (B) female mosquitoes were housed separately and analyzed independently. No significant change to survival was observed out to 28 days when the experiment was ended; however, the P value for infected female mosquitoes is 0.07, indicating that there is possibly a fitness benefit for females colonized by *C*. *fasciculata*. Survival curves were compared using a Log-rank (Mantel-Cox) test. Data representative of and pooled from two independent replicates.
(TIF)

S2 Fig RNAseq reads mapping to *Ae*. *aegypti* from mosquito hindgut samples. The light shaded bars are uninfected hindguts and the darker bars are infected hindguts. The percentages indicate the fraction of reads Kallisto mapped to *Ae*. *aegypti* transcriptome version AaegL5.1 in each replicate.
(TIF)

S3 Fig Relative transcript abundance of selected *C*. *fasciculata* genes by qRT-PCR. Selected genes (Ank, CFAC1_300013200; PP2C, CFAC1_060021100; TPR, CFAC1_090020800; Craltrio, CFAC1_190022700; hyp544, CFAC1_270054400; GP63-800, CFAC1_040011800) are single-copy where specific qRT-PCR primers could be designed. Positive and negative bars indicate enrichment in adherent and swimming cells, respectively. Dark and light shading indicate RNAseq enrichment in adherent or swimming cells, respectively. There is a correspondence between positive bars and RNAseq enrichment in adherent cells, and negative bars and RNAseq enrichment in swimming cells, which verifies the RNAseq results. Data averaged from three replicates. Error bars show the standard deviation.
(TIF)

S4 Fig Relative transcript abundance of selected *C*. *fasciculata gp63* genes by qRT-PCR. Specific qRT-PCR primers were designed for selected *gp63* genes (CFAC1_040011800, CFAC1_270047600, CFAC1_150013400, CFAC1_040011600, CFAC1_040011700, CFAC1_040012000, CFAC1_270047200). Positive and negative bars indicate enrichment in adherent and swimming cells, respectively. Dark and light shading indicate RNAseq enrichment in adherent or swimming cells, respectively. The darkest bar indicates enrichment in the hindgut sample. There is a correspondence between positive bars and RNAseq enrichment in adherent cells, and negative bars and RNAseq enrichment in swimming cells, which verifies the RNAseq results. Data averaged from three replicates. Error bars show the standard error.
(TIF)

S5 Fig GSEA of putative amino acyl transfer RNA biosynthesis KEGG pathway. The left panel shows a heat map of enrichment in adherent (Adh) or swimming (Swim) forms for the three individual replicates (bottom). TriTrypDB gene identifiers, and where applicable, 3-letter amino acid codes are shown. Red color indicates transcript enrichment in adherent form and blue indicates enrichment in swimming form. The 23 genes above the red dashed line (GSEA software threshold) are considered as significantly contributing to the enrichment of the set. The right panel shows the enrichment plot generated by the GSEA software along with the Normalized Enrichment Score (NES) and False Discovery Rate (FDR) indicated for this gene set. A positive NES indicates enrichment in adherent form. The gray shaded area shows a rank order of the genes based on their enrichment in adherent (left, red) or swimming forms (right, blue). The black vertical lines indicate the position of the 31 members of the set within the rank order. The density of lines on the left indicates the enrichment of genes in adherent form. The green line indicates the enrichment profile generated by the software.
(TIF)

S1 Movie Time-lapse of adherent cell growth. Adherent cells growing in culture for 24 hours. This movie contains images shown in Fig 3A. Images were collected every 2 minutes and 30 seconds as indicated in the time stamp in the upper right corner.
(MOV)

S2 Movie Time-lapse of adherent cell growth. Another field of the experiment shown in Fig 3A and S1 Movie. Images were collected every 2 minutes and 30 seconds as indicated in the time stamp in the upper right corner.
(MOV)

S3 Movie Time-lapse of cell-division in rosettes. Rosette growing in culture for 4 hours. This movie contains images shown in Fig 3B. Images were collected approximately every 2 minutes as indicated in the time stamp in the upper right corner.
(MOV)

S1 Table Transcripts upregulated in cultured adherent *C*. *fasciculata*. Gene IDs from TriTrypDB for *C*. *fasciculata* transcripts significantly upregulated in cultured adherent cells relative to cultured swimming cells (LFC>1, p<0.01) across six samples (three cultured swimming and three cultured adherent). Also shown is the annotation from TriTrypDB (Product Description). Genes comprise Group 2 of Fig 6A and are listed in order of accession number.
(XLSX)

S2 Table Transcripts downregulated in cultured adherent *C*. *fasciculata*. Gene IDs from TriTrypDB for *C*. *fasciculata* transcripts significantly downregulated in cultured adherent cells relative to cultured swimming cells (LFC>1, p<0.01) across six samples (three cultured swimming and three cultured adherent). Also shown is the annotation from TriTrypDB (Product Description). Genes comprise Group 1 of Fig 6A and are listed in order of accession number.
(XLSX)

S3 Table *C*. *fasciculata* transcripts upregulated in mosquito hindgut. Gene IDs from TriTrypDB for *C*. *fasciculata* transcripts significantly upregulated in parasites isolated from infected hindguts compared to cultured swimming cells across nine samples (three cultured swimming, three cultured adherent, and three from infected hindguts). Shown is the annotation from TriTrypDB (Product Description). Genes are listed in order of accession number. These genes are described in Fig 6D.
(XLSX)

S4 Table *C*. *fasciculata* transcripts upregulated in mosquito hindgut and in cultured adherent cells. Gene IDs from TriTrypDB for *C*. *fasciculata* transcripts significantly upregulated in parasites isolated from infected hindguts and cultured adherent cells compared to cultured swimming cells in our nine-sample analysis. Shown is the annotation from TriTrypDB (Product Description). Genes are listed in order of accession number. These genes are described in Fig 6D.
(XLSX)

S5 Table GSEA lists analyzed. 134 gene sets and associated KEGG pathway number available for *C*. *fasciculata* genes on TriTrypDB. Two additional gene sets, GP63 proteins and putative Paraflagellar Rod Proteins were manually generated. 96 lists had sufficient numbers of members to be included in the analysis after restricting the list based on presence in the dataset (Yes). Lists in italics were excluded due to an insufficient number of genes with data in the dataset. Total number of genes in each set are shown as well as the number of genes per set contributing to the analysis. Sets not analyzed are listed as “No” with number of genes indicated as “na”.
(XLSX)

S6 Table *Ae*. *aegypti* transcripts upregulated in *C*. *fasciculata* infected hindguts. (XLSX)

S7 Table *Ae*. *aegypti* transcripts downregulated in *C*. *fasciculata* infected hindguts. (XLSX)

S8 Table Primers. (XLSX)

S1 File Gene sets with member genes for GSEA analysis. (TXT)

S2 File Tab delimited *C*. *fasciculata* expression data in GCT format for GSEA analysis. (TXT)
