# Supplementary figures and images for: Dramatic changes in gene expression in different forms of Crithidia fasciculata reveal potential mechanisms for insect-specific adhesion in kinetoplastid parasites
Source: PLoS Negl Trop Dis. 2019 Jul 29;13(7):e0007570. doi: 10.1371/journal.pntd.0007570 (PMC6687205; doi:10.1371/journal.pntd.0007570)

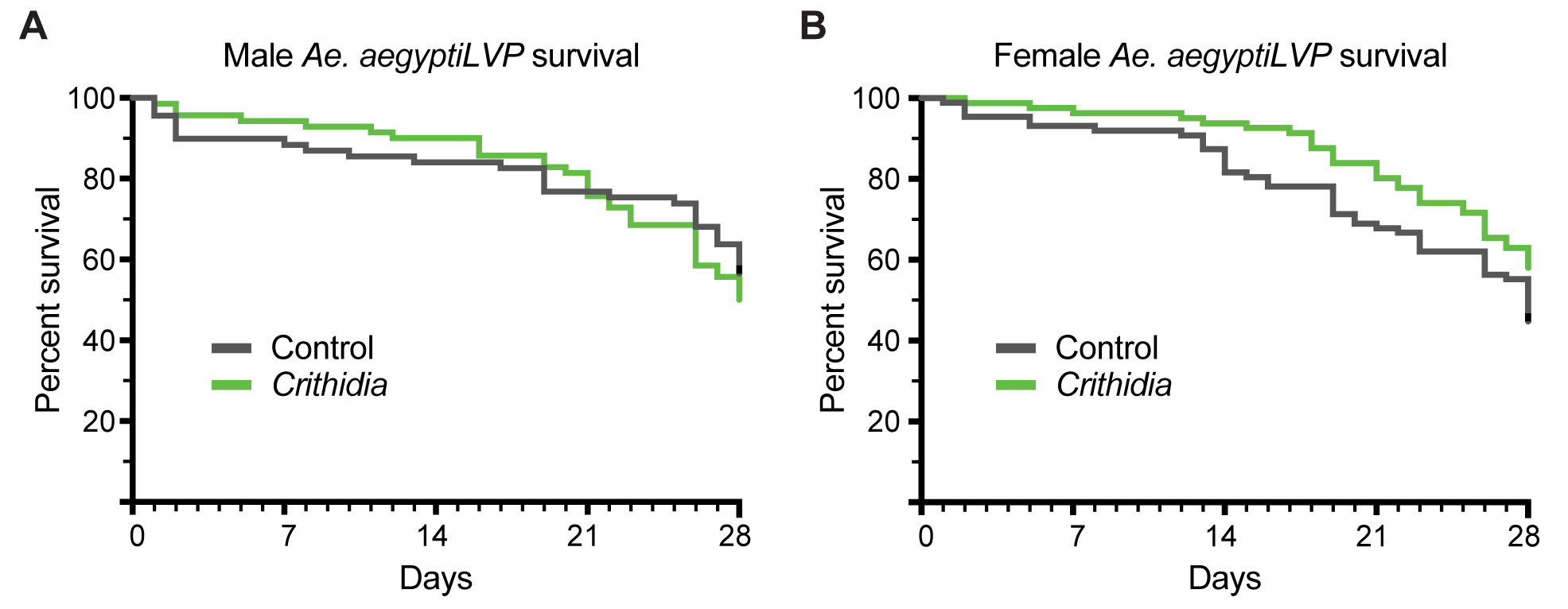

Supplement: S1 Fig — Kaplan-Meier survival plots of Ae. aegypti following overnight feeding with C. fasciculata in a sucrose meal. Under these conditions virtually all mosquitoes are infected. (A) Male and (B) female mosquitoes were housed separately and analyzed independently. No significant change to survival was observed out to 28 days when the experiment was ended; however, the P value for infected female mosquitoes is 0.07, indicating that there is possibly a fitness benefit for females colonized by C. fasciculata. Survival curves were compared using a Log-rank (Mantel-Cox) test. Data representative of and pooled from two independent replicates. (TIF) [file pntd.0007570.s001.tif]

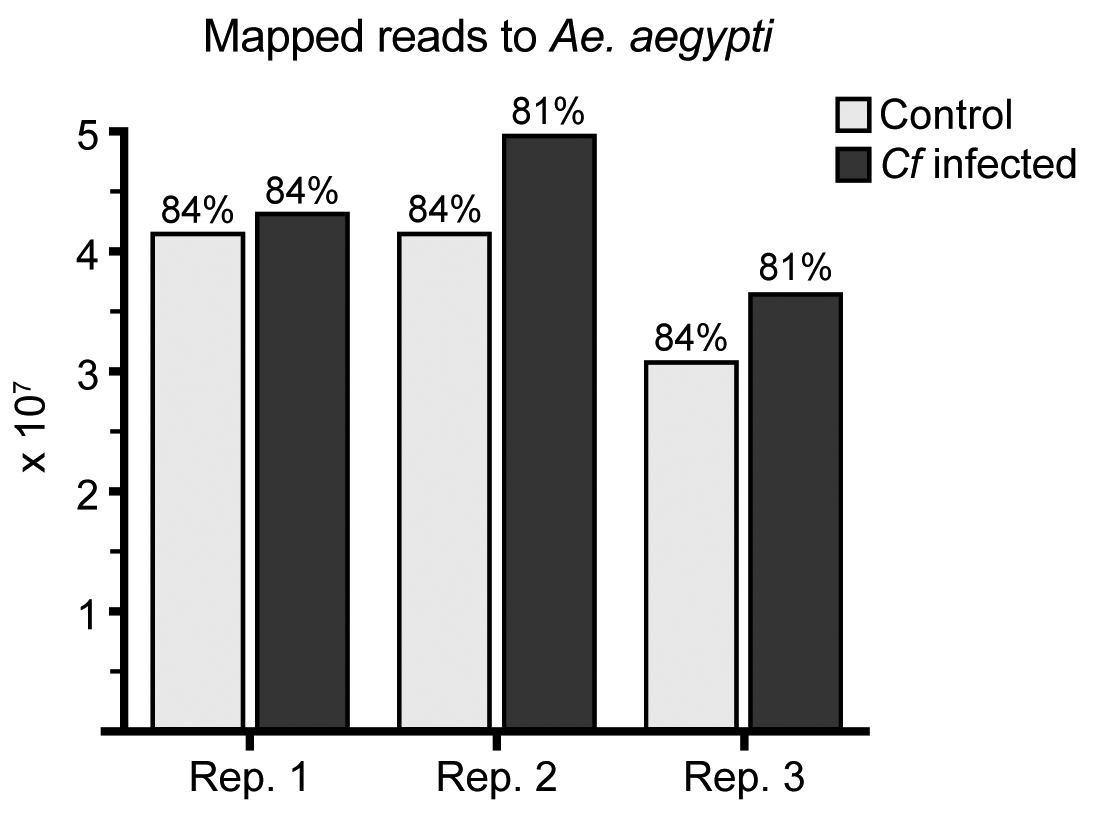

Supplement: S2 Fig — The light shaded bars are uninfected hindguts and the darker bars are infected hindguts. The percentages indicate the fraction of reads Kallisto mapped to Ae. aegypti transcriptome version AaegL5.1 in each replicate. (TIF) [file pntd.0007570.s002.tif]

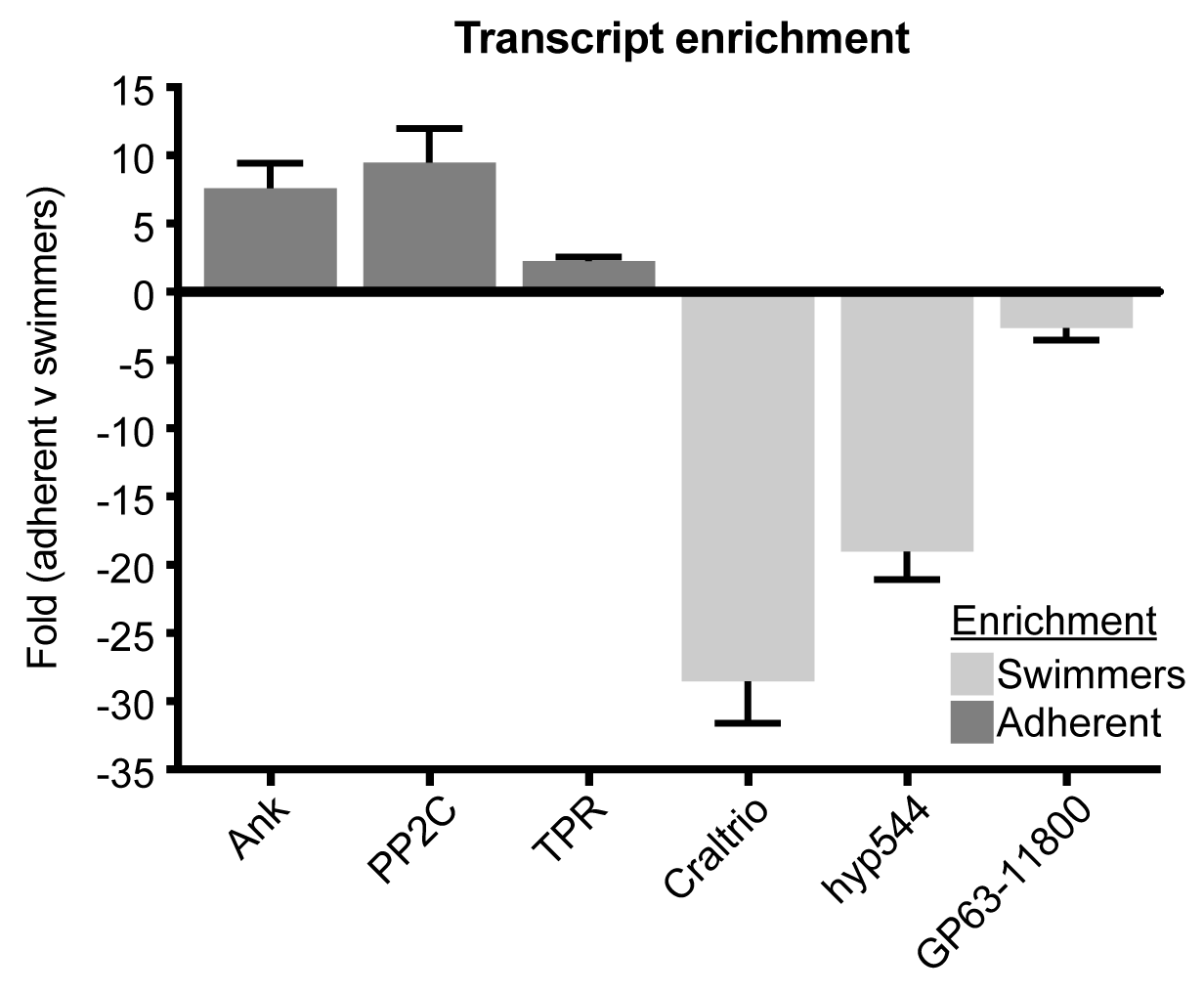

Supplement: S3 Fig — Selected genes (Ank, CFAC1_300013200; PP2C, CFAC1_060021100; TPR, CFAC1_090020800; Craltrio, CFAC1_190022700; hyp544, CFAC1_270054400; GP63-800, CFAC1_040011800) are single-copy where specific qRT-PCR primers could be designed. Positive and negative bars indicate enrichment in adherent and swimming cells, respectively. Dark and light shading indicate RNAseq enrichment in adherent or swimming cells, respectively. There is a correspondence between positive bars and RNAseq enrichment in adherent cells, and negative bars and RNAseq enrichment in swimming cells, which verifies the RNAseq results. Data averaged from three replicates. Error bars show the standard deviation. (TIF) [file pntd.0007570.s003.tif]

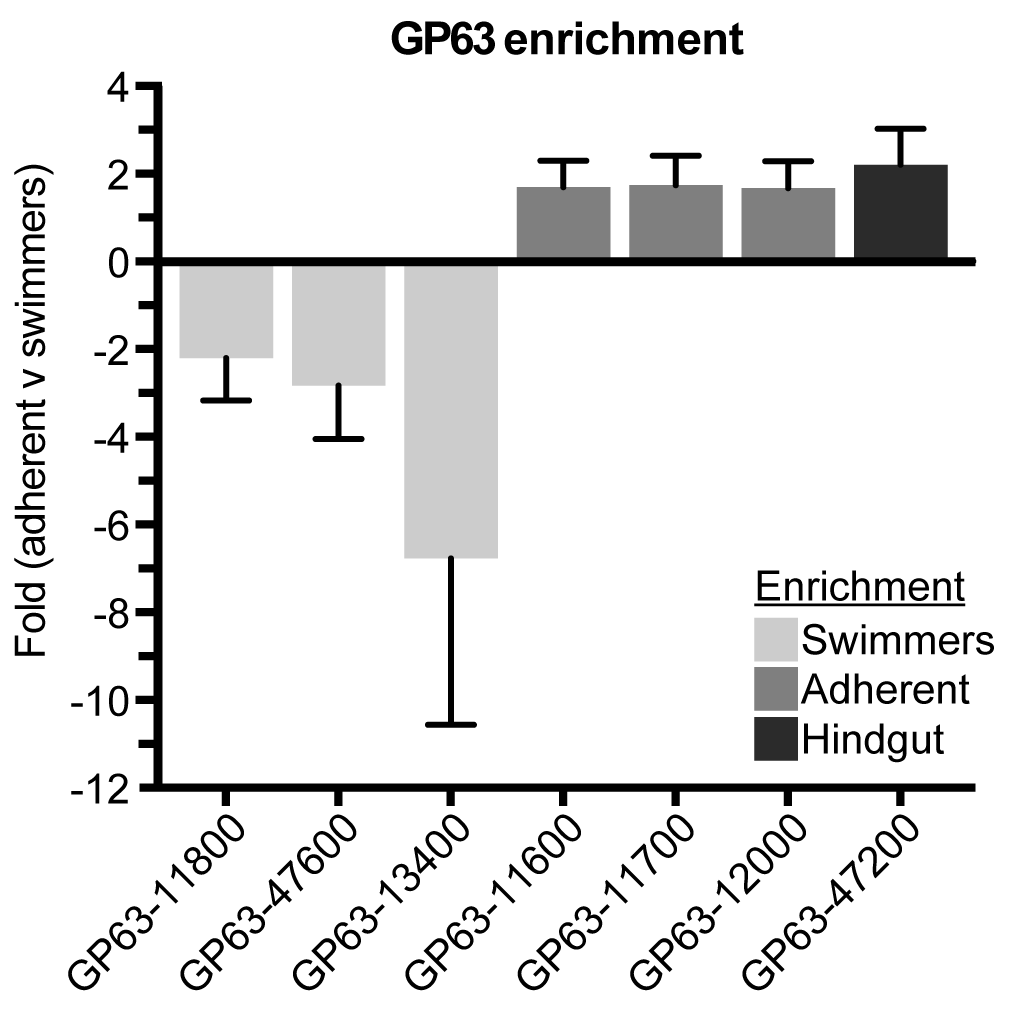

Supplement: S4 Fig — Specific qRT-PCR primers were designed for selected gp63 genes (CFAC1_040011800, CFAC1_270047600, CFAC1_150013400, CFAC1_040011600, CFAC1_040011700, CFAC1_040012000, CFAC1_270047200). Positive and negative bars indicate enrichment in adherent and swimming cells, respectively. Dark and light shading indicate RNAseq enrichment in adherent or swimming cells, respectively. The darkest bar indicates enrichment in the hindgut sample. There is a correspondence between positive bars and RNAseq enrichment in adherent cells, and negative bars and RNAseq enrichment in swimming cells, which verifies the RNAseq results. Data averaged from three replicates. Error bars show the standard error. (TIF) [file pntd.0007570.s004.tif]

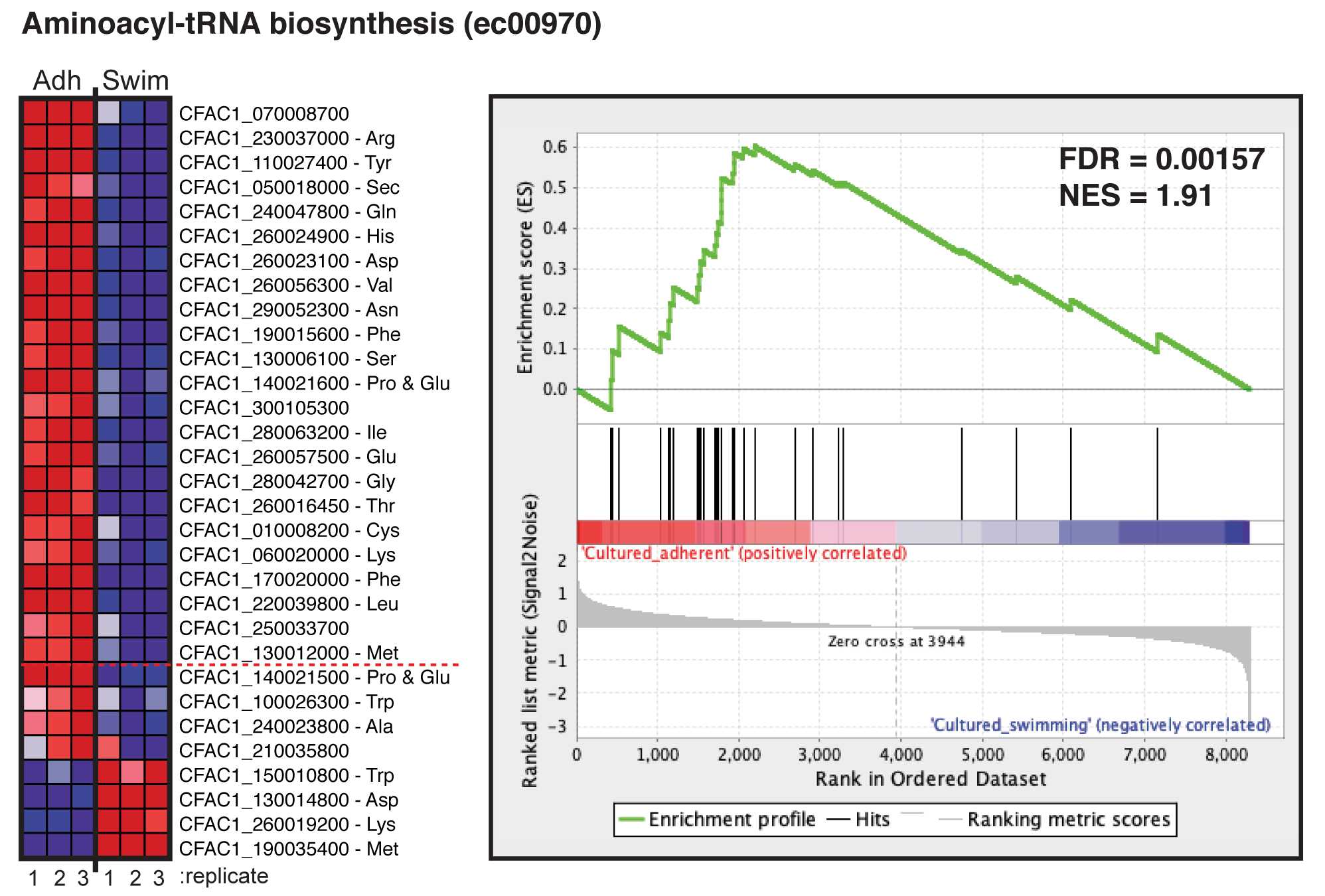

Supplement: S5 Fig — The left panel shows a heat map of enrichment in adherent (Adh) or swimming (Swim) forms for the three individual replicates (bottom). TriTrypDB gene identifiers, and where applicable, 3-letter amino acid codes are shown. Red color indicates transcript enrichment in adherent form and blue indicates enrichment in swimming form. The 23 genes above the red dashed line (GSEA software threshold) are considered as significantly contributing to the enrichment of the set. The right panel shows the enrichment plot generated by the GSEA software along with the Normalized Enrichment Score (NES) and False Discovery Rate (FDR) indicated for this gene set. A positive NES indicates enrichment in adherent form. The gray shaded area shows a rank order of the genes based on their enrichment in adherent (left, red) or swimming forms (right, blue). The black vertical lines indicate the position of the 31 members of the set within the rank order. The density of lines on the left indicates the enrichment of genes in adherent form. The green line indicates the enrichment profile generated by the software. (TIF) [file pntd.0007570.s005.tif]
